# Computational fluid dynamics to simulate stenotic lesions in coronary end-to-side anastomosis

**DOI:** 10.1093/icvts/ivaf013

**Published:** 2025-01-31

**Authors:** Kenichi Kamiya, Shinya Terada, Yukihiro Nagatani, Yuji Matsubayashi, Kohei Suzuki, Shohei Miyazaki, Hiroki Matsui, Shota Takano, Susumu Nakata, Yoshiyuki Watanabe, Tomoaki Suzuki

**Affiliations:** Department of Cardiovascular Surgery, Shiga University of Medical Science, Otsu, Shiga, Japan; Graduate School of Medicine, Shiga University of Medical Science, Otsu, Shiga, Japan; Department of Radiology, Shiga University of Medical Science, Otsu, Shiga, Japan; Department of Cardiovascular Surgery, Shiga University of Medical Science, Otsu, Shiga, Japan; Cardio Flow Design Inc, Tokyo, Japan; Cardio Flow Design Inc, Tokyo, Japan; Graduate School of Information Science and Engineering, Ritsumeikan University, Kusatsu, Shiga, Japan; Graduate School of Information Science and Engineering, Ritsumeikan University, Kusatsu, Shiga, Japan; College of Information Science and Engineering, Ritsumeikan University, Kusatsu, Shiga, Japan; Department of Radiology, Shiga University of Medical Science, Otsu, Shiga, Japan; Department of Cardiovascular Surgery, Shiga University of Medical Science, Otsu, Shiga, Japan

**Keywords:** computational fluid dynamics, coronary artery bypass grafting, end-to-side anastomosis, *ex vivo* study

## Abstract

**OBJECTIVES:**

End-to-side anastomosis is common in coronary artery bypass grafting, although restrictive suturing can narrow the anastomosis. We evaluated *ex vivo* end-to-side models by numerically simulating fluid dynamics to compare various degrees of stenotic anastomoses to predict haemodynamic effects.

**METHODS:**

A carotid artery was grafted via an end-to-side anastomosis onto the left anterior descending artery of a porcine heart, with liquid silicone injected into the vessels. The end-to-side image was acquired via multidetector computed tomography for reference, and models of longitudinal shortening and bilateral narrowing were created with 25%, 50%, 75%, along with 90%, and 100% stenosis in the native coronary artery. Haemodynamics were analysed using computational fluid dynamics simulations to calculate streamlines, wall shear stress and oscillatory shear index.

**RESULTS:**

In the reference model, the graft inflow impinged on the floor of the native artery, creating a recirculating vortex and a high oscillatory shear index region near the heel. As the graft flow angle increased with longitudinal stenosis, bilateral stenosis generated helical flow near the lateral wall of the native artery, worsening with increased stenosis. At 75% stenosis, both longitudinal shortening and bilateral narrowing caused abnormal flow separation, with low wall shear stress and high oscillatory regions forming distal to the toe of the anastomosis.

**CONCLUSIONS:**

Computational fluid dynamics modelling predicts that end-to-side anastomoses with 75% longitudinal or bilateral stenosis are at a risk of intimal hyperplasia causing graft failure, while anastomotic stenosis <50% indicates acceptable haemodynamics. Future studies should explore long-term clinical outcomes with suboptimal surgical anastomotic construction.

**CLINICAL REGISTRATION NUMBER:**

Not applicable.

## Graphical Abstract

**Figure:**
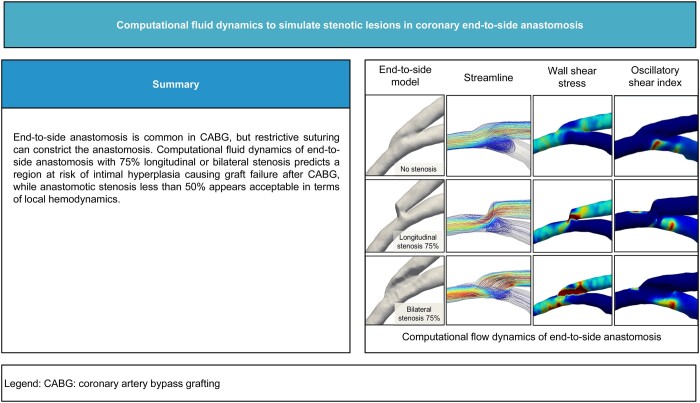

## INTRODUCTION

Coronary artery bypass grafting (CABG) is effective for alleviating ischaemic heart disease; however, its long-term benefits depend on graft patency [1]. The distal end-to-side anastomosis is a common graft configuration, although early failures often stem from surgical errors and thrombosis, while later failures result from atherosclerosis and intimal hyperplasia [2, 3].

A frequent technical error in an end-to-side anastomosis is suture constriction, or the ‘purse-stringing effect’, caused by deep bites or widely spaced stiches in the continuous suture [4, 5]. To mitigate this, small stitches at the heel and toe (proximal and distal ends) of the anastomosis are recommended, whereas deeper bites along the sides are considered less problematic [6]. While the importance of precise operative techniques for constructing an impeccable anastomosis in CABG has been highlighted, limited evidence exists regarding the effect of suboptimal configurations of end-to-side anastomoses.

Computational fluid dynamics (CFD) is widely used to simulate dynamic parameters such as flow velocity, wall shear stress (WSS) and flow distribution visualized on streamlines [3, 7]. Experimental studies suggest that haemodynamic factors, including disturbed flow or flow stagnation, especially at the distal anastomosis, may contribute to intimal hyperplasia, eventually leading to graft failure [3, 8–10]. Vascular endothelial cells exposed to excessively low or high shear stress may develop structural and functional abnormalities, with areas of low-magnitude and high-oscillatory WSS being most prone to intimal hyperplasia [11]. Currently, CFD simulations are potentially the most efficient approach for bridging the gap between experimental findings and clinical applications. Several CFD-based studies have investigated anatomical factors influencing graft failure in CABG, including graft-to-host artery diameter ratios, graft configurations and graft types [9, 12]. However, limited simulation data exist on clinically relevant aspects, including various deleterious effects of anastomotic stenosis and the correlation between stenotic severity and graft failure risk. Therefore, this study aimed to simulate the fluid dynamics of end-to-side anastomoses using an *ex vivo* porcine heart, modelling various stenotic conditions related to imperfect surgical techniques, and predict haemodynamic effects of stenotic lesions that may affect graft patency.

## MATERIALS AND METHODS

### Study overview

We analysed coronary end-to-side anastomoses by comparing various stenosis models using CFD technique. An *ex vivo* end-to-side anastomosis was created using a porcine heart and carotid artery, and liquid silicone was injected into the vessels. Images of the silicone cast model were acquired using a multidetector CT scanner in an ultra-high-resolution mode (spatial resolution: 0.25 mm). The end-to-side anastomosis image (reference) was segmented, and various stenosis models were generated using image-morphing techniques in computer graphics. Computational simulations were performed for 14 models—the original anastomosis model (0% stenosis) and 6 models involving three degrees of stenosis (25%, 50% and 75%) combined with two different patterns of anastomotic stenosis (longitudinal shortening and bilateral narrowing), with each model paired with two native coronary artery stenosis grades (90% and 100%). Blood flow simulations were performed using physiological boundary conditions applied to the simulation domain. Based on the calculated results, haemodynamic effects in anastomosis regions predicting graft failure were evaluated by comparing various types and degrees of stenosis. The comprehensive methodology, including image acquisition, postprocessing, and computational simulations, is described in the Supplementary Material, Methods.

### Ethics approval

The explanted porcine heart and carotid artery specimens were obtained from a dedicated distributor (ATSCO Inc, TX, USA). These tissues were harvested at local abattoirs, with the by-products commercially supplied for surgical training and educational purposes. Therefore, our institutional review board waived the ethical approval for this study.

### *Ex vivo* porcine coronary anastomosis model

**Figure 1:**
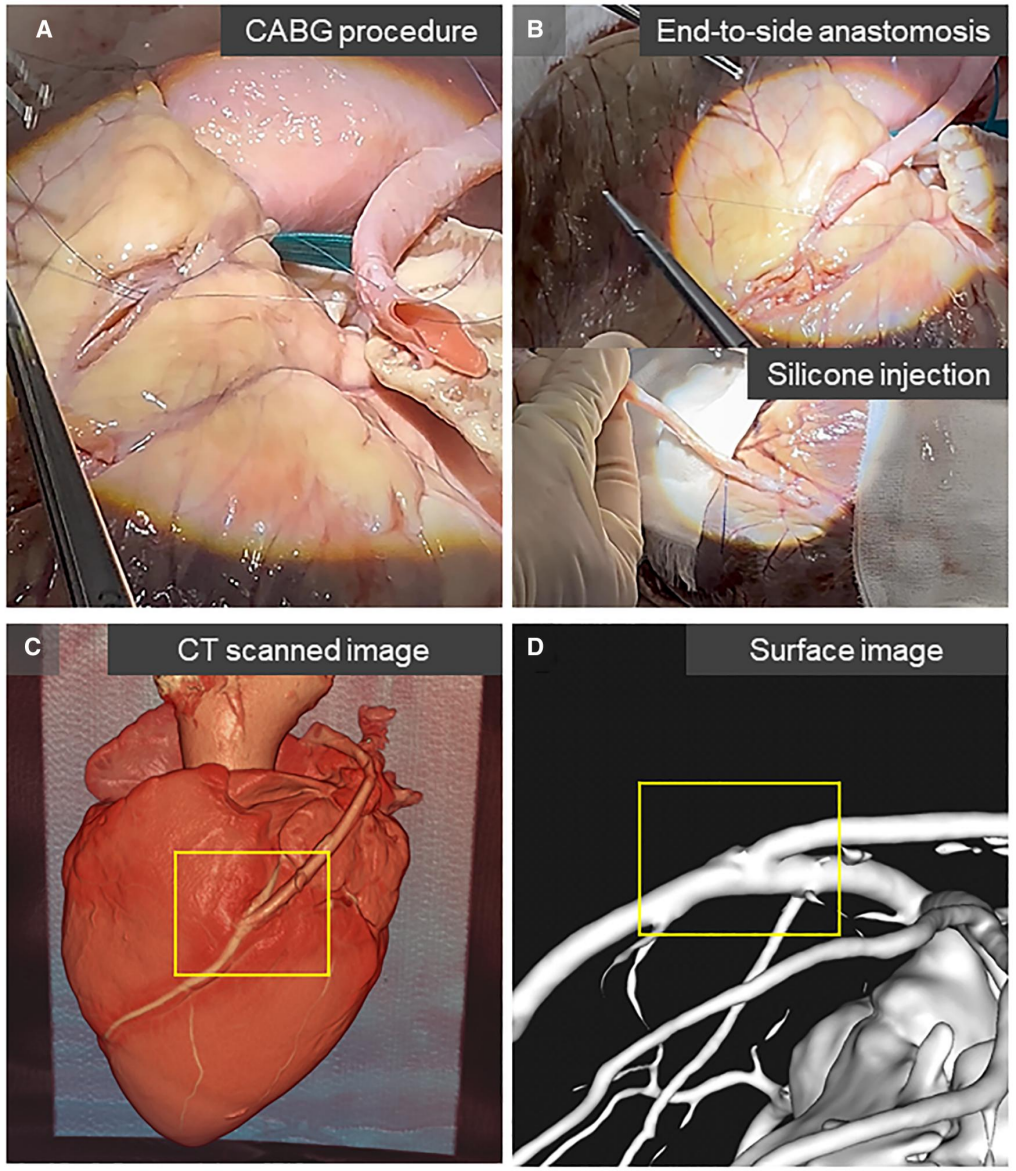
Image generation of an *ex vivo* end-to-side model. (**A**) A porcine carotid artery was grafted onto a porcine coronary artery. (**B**) A liquid silicone was injected into the graft and native coronary artery. (**C**) Three-dimensional image of computed tomography (CT) data showing the bypassed graft to the left anterior descending artery (yellow box). (D) A generated surface image of the end-to-side anastomosis (yellow box). CABG: coronary artery bypass grafting.

As illustrated in Fig. 1, the porcine carotid artery was grafted onto the midportion of the left anterior descending artery (LAD) of the porcine heart. The target native coronary artery, with proximal and distal diameters of 2.8 mm and 3.2 mm, respectively, was incised using a scalpel blade and Potts scissors. The arteriotomy was extended to 10 mm, and the graft vessel end was trimmed to match this length. A distal end-to-side anastomosis was performed using a continuous running 7–0 polypropylene suture.

### Creation of silicone cast

A two-component liquid silicone suspension (ELASTOSIL P-7684/60A/B, Wacker-Chemie-AG, Munich, Germany) was thoroughly mixed with a solidifier (Fig. 1), and injected into the graft, ensuring that the coronary artery was filled. The cast was left undisturbed for 2 h until it solidified.

### Image data acquisition of the CABG model

The grafted porcine heart was positioned with the anastomosis facing upward for computed tomography (CT) scanning. Image data were acquired from the silicone cast model using a 160-row multidetector CT scanner (Aquilion Precision, Canon Medical Systems, Otawara, Tochigi, Japan) in ultra-high-resolution mode with a spatial resolution of 0.25 mm.

### Image processing for surface segmentation

Image data in Digital Imaging and Communications in Medicine format were transferred into Vesalius3D software (version 2.12; PS-tech, Amsterdam, Netherlands), and the mask volume of the end-to-side anastomosis was extracted (Fig. 1).

### Generation of stenotic end-to-side anastomosis models and meshing

**Figure 2:**
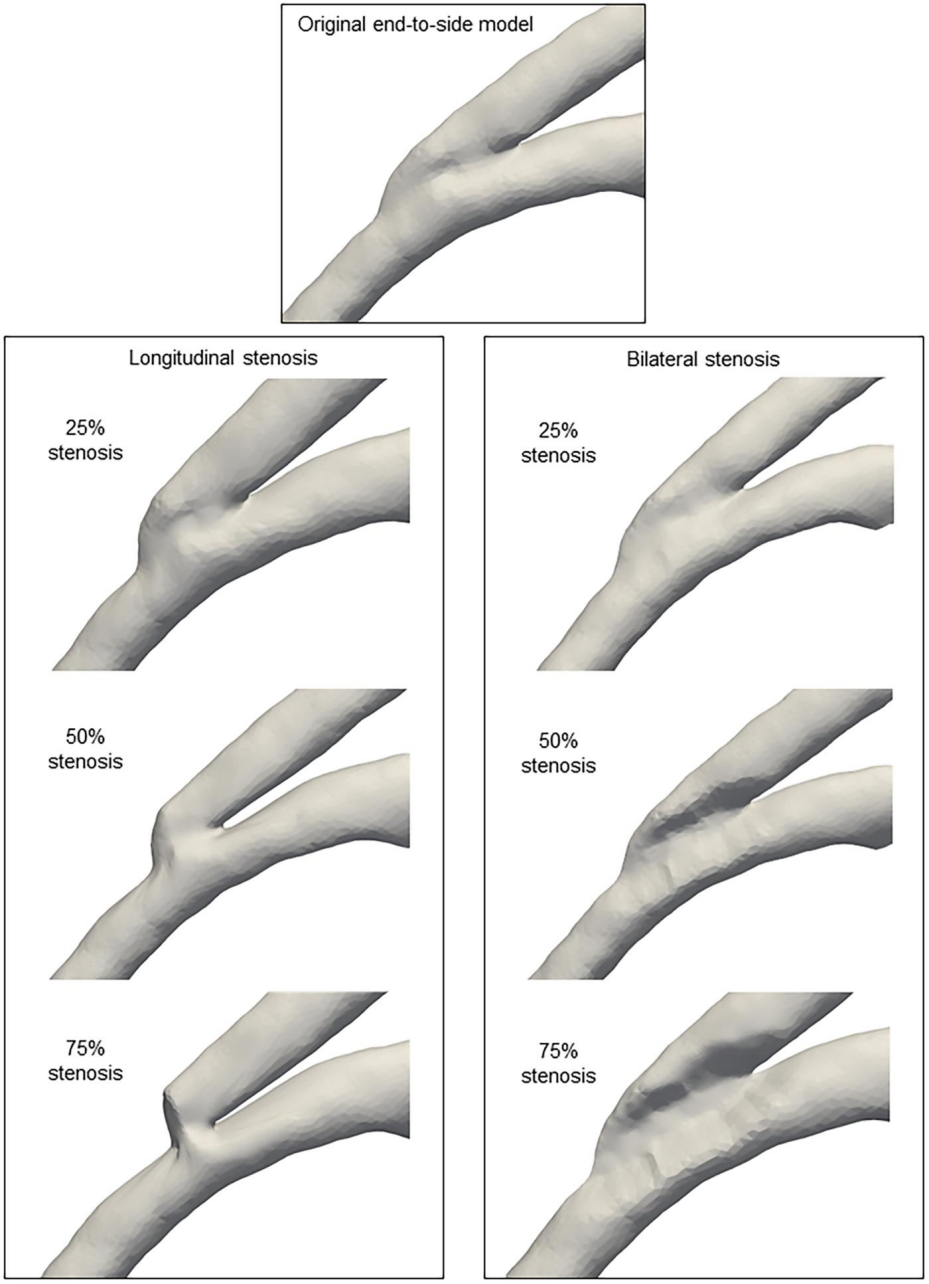
Longitudinal and bilateral stenosis models of the end-to-side anastomosis. The stenosis models were derived from the original end-to-side model. The longitudinal model shows inward movement of the toe and heel edges, while the bilateral model also includes inward movement of the near and far edges.

Various stenosis models were created using a shape-blending technique (Fig. 2 and Supplementary Material, Fig. S1). The surface data of the original end-to-side anastomosis served as the reference model. Target stenosis models were generated using open-source Blender software (version 3.2.2, Blender Foundation, Amsterdam, Netherlands). For longitudinal stenosis, both the toe and heel edges were transformed toward the centre of the anastomosis, whereas transverse stenosis was simulated by narrowing the sides. Models representing 25%, 50% and 75% stenoses were artificially generated. Native coronary lesions (90% and 100% stenosis) were created using the same technique. Computational meshes were generated using ANSYS-ICEM CFD 16.0 (ANSYS Japan, Tokyo, Japan), with each mesh comprising over 2 million elements. To enhance accuracy near the wall, three prism layers were added to refine the mesh.

### Computational simulation

The velocity and pressure of blood flow in the simulation domain were calculated based on boundary conditions that reflected physiological aortic flow volumes, pressure waves and peripheral coronary impedances. The results were postprocessed to visualize and quantify the haemodynamic parameters. Individual simulation results were summarized and visually compared across various degrees of anastomotic and native coronary stenoses. Details of the boundary conditions and calculations of the haemodynamic parameters are provided in the Supplementary Material, Methods.

#### Boundary conditions

**Figure 3:**
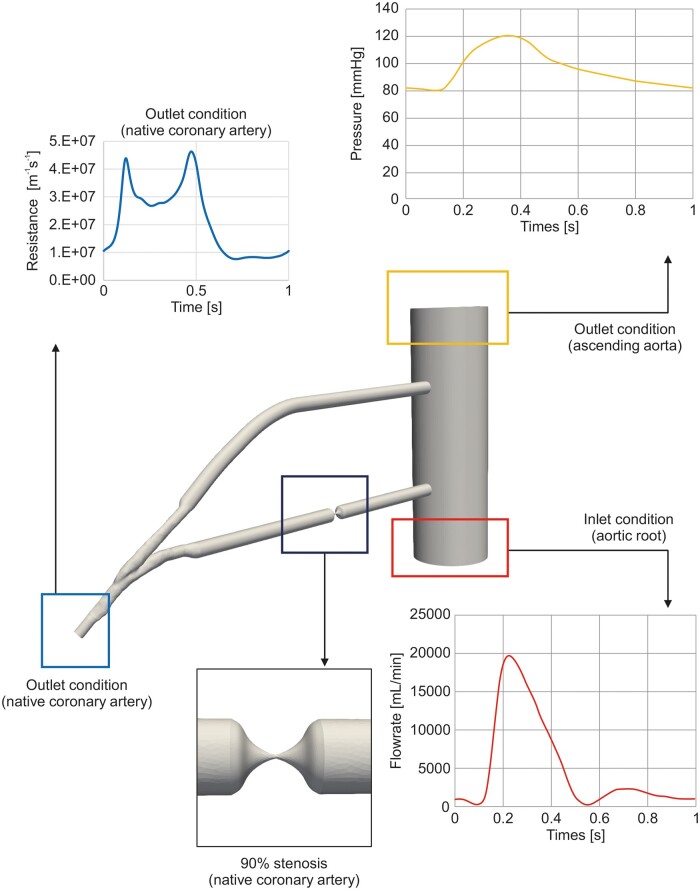
Boundary conditions for computational fluid dynamics analysis in the 90% native coronary stenosis models. Inlet flow rate at the aortic root (red box), outlet pressure at the ascending aorta (yellow box) and time-varying impedance of the coronary artery outlet (blue box) were applied to simulate flow in the anastomosed region.

To simulate flow in the anastomosed region, a simulation model was developed that allowed for the explicit specification of time-varying flow rates and pressures as boundary conditions (Fig. 3). In this model, the inlet boundary condition at the aortic root was set to a flow rate of 5.0 l/min with a pulsatile waveform, whereas the outlet condition at the ascending aorta was maintained at an average pressure of 96 mmHg [13, 14]. Time-varying impedance, reflecting physiological peripheral resistance during one cardiac cycle, was applied to the coronary artery outlet boundary of the LAD to simulate peripheral vascular beds. The vessel walls, including the extended boundary walls, were modelled as rigid structures.

#### Pulsatile flow analysis

The finite volume method was employed to solve the Navier–Stokes and continuity equations using the open-source CFD software program, OpenFOAM version 8.0 (OpenFOAM Foundation, London, UK). Blood was treated as an incompressible Newtonian fluid with a density of 1060 kg/m^3^ and a viscosity of 0.004 Pa·s. Each simulation was run for one cardiac cycle (1 s) to obtain a periodic solution representing physiologic pulsatile flow. The calculated results were postprocessed to visualize and quantify the haemodynamic parameters using ParaView 5.1.1 (Kitware, Inc., New York, USA).

#### Anastomosis evaluations of near-wall haemodynamics

From the computational results, we evaluated the visualized blood flow patterns (‘streamlines’) and analysed velocity vectors. Near-wall haemodynamics were calculated, including WSS (the tangential frictional force exerted on the vascular inner surface), its fluctuations as the oscillatory shear index (OSI) [15, 16] and the relative residence time (RRT) [17] as a metric of disturbed blood flow.

## RESULTS

### Characteristics of the stenotic end-to-side models

The morphological characteristics of the end-to-side anastomosis, including the perimeter and area of the stenosis models, are shown in Supplementary Material, Table S1.

### Streamlines in the end-to-side models with 90% native coronary stenosis

**Figure 4:**
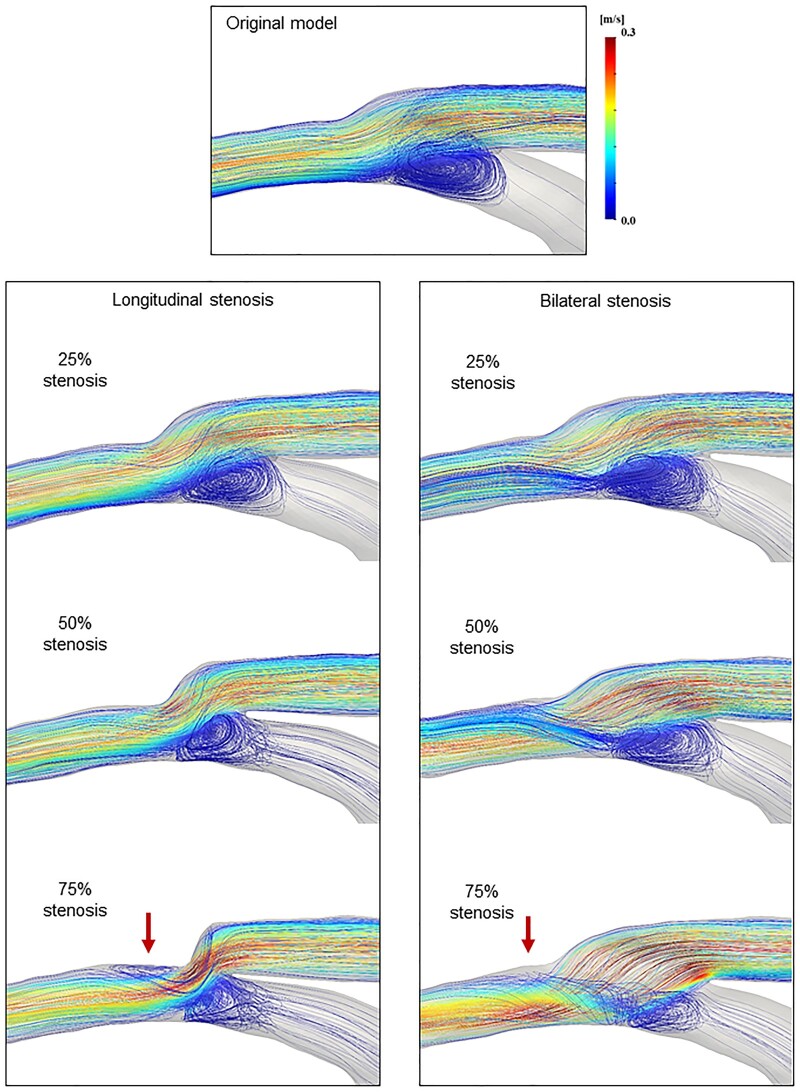
Streamlines in the end-to-side models with longitudinal or bilateral stenosis. The original model shows a vortex at the heel, arterial floor impingement and distal laminar flow. With longitudinal stenosis, a narrower anastomosis created a steeper flow angle. Conversely, bilateral stenosis generated helical flow along the lateral wall. In both 75% stenotic patterns, flow impinged on the native artery floor, leading to flow separation at the toe (red arrows).

The streamlines generated by the original model and various stenosis models are shown in Fig. 4 and Supplementary Material, Figs S2, S3 and S6. The calculated flow rates corresponding to different anastomotic morphologies are shown in Supplementary Material, Tables S2 and S3.

In the original model, which exhibited no anastomotic stenosis, the graft flow entering the native coronary artery primarily skewed downward at the toe end of the graft but maintained laminar flow further downstream from the anastomosis. Concurrently, a stagnation zone formed within the native artery, where a recirculation zone (‘vortex’) developed at the heel due to the interaction between the graft flow and the relatively slow flow from the largely occluded proximal artery. This vortex was accompanied by a flow separation zone on the native coronary artery bed, where the graft flow impinged. The location of this zone oscillated with the vortex size during the diastolic phase.

With longitudinal stenosis, the angle of flow at the anastomosis site increased with the degree of stenosis. At 75% stenosis, additional flow separation occurred at the toe, with a reversed-flow region emerging downstream of the anastomosis, whereas the vortex length at the heel was reduced. In the bilateral stenosis model, the graft inflow through the anastomosis impinged on the floor of the native coronary artery, creating a recirculating vortex at the heel. Beyond the central inflow, a spiral pattern of motion (‘helical flow’) developed along the lateral wall, particularly in the 50% and 75% stenosis. At 75% stenosis, flow separation at the toe appeared in the reverse flow region, whereas the vortex size at the heel was minimized. To further illustrate pulsatile blood flow, streamlines and velocity vectors are shown in Supplementary Material, Videos S1 and S2.

### The WSS and OSI values of the anastomosis with 90% native coronary stenosis

**Figure 5:**
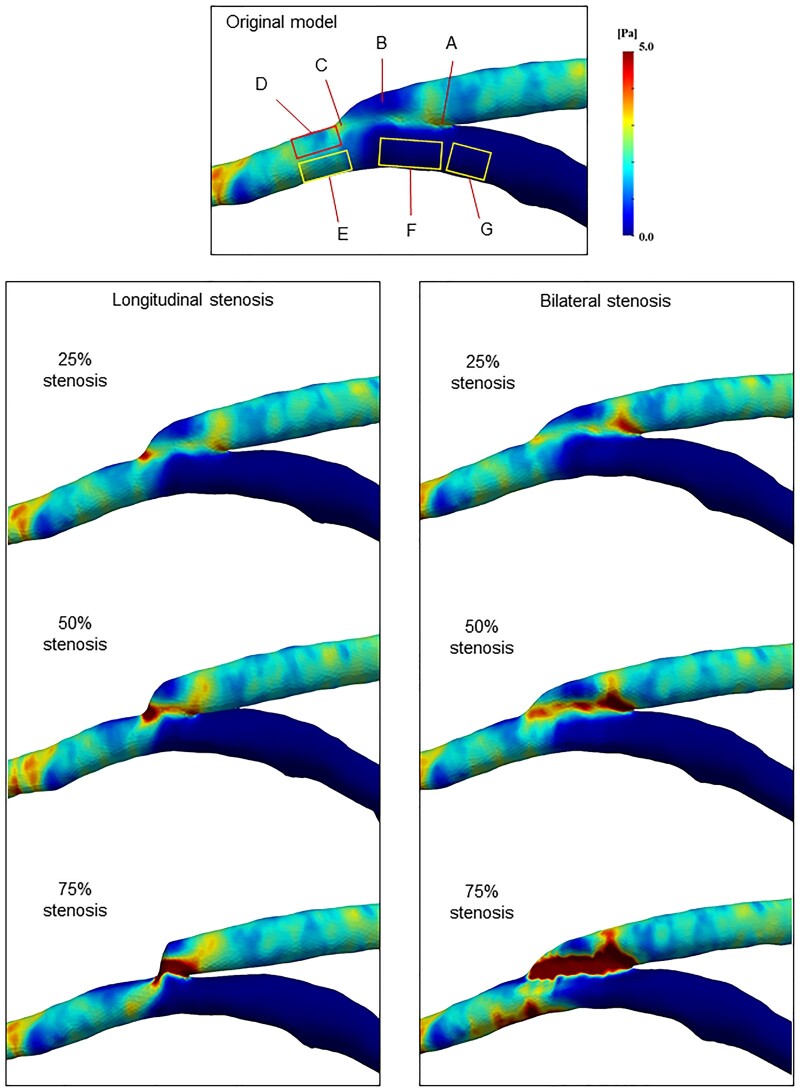
Wall shear stress (WSS) distributions around the end-to-side anastomosis with longitudinal or bilateral stenosis. In the original model, high-WSS areas were observed at the heel and toe (region A, C), whereas low-WSS areas were at the roof of the graft, floor of the heel and upstream in the native artery (region B, F and G). With longitudinal stenosis, the maximum WSS occurred at the toe, whereas bilateral stenosis showed high WSS at the heel. Both stenotic patterns with 75% stenosis had low WSS at the upper side of the distal toe (region D), whereas the lower side of the distal toe (region E) showed higher WSS in bilateral stenosis.

**Figure 6:**
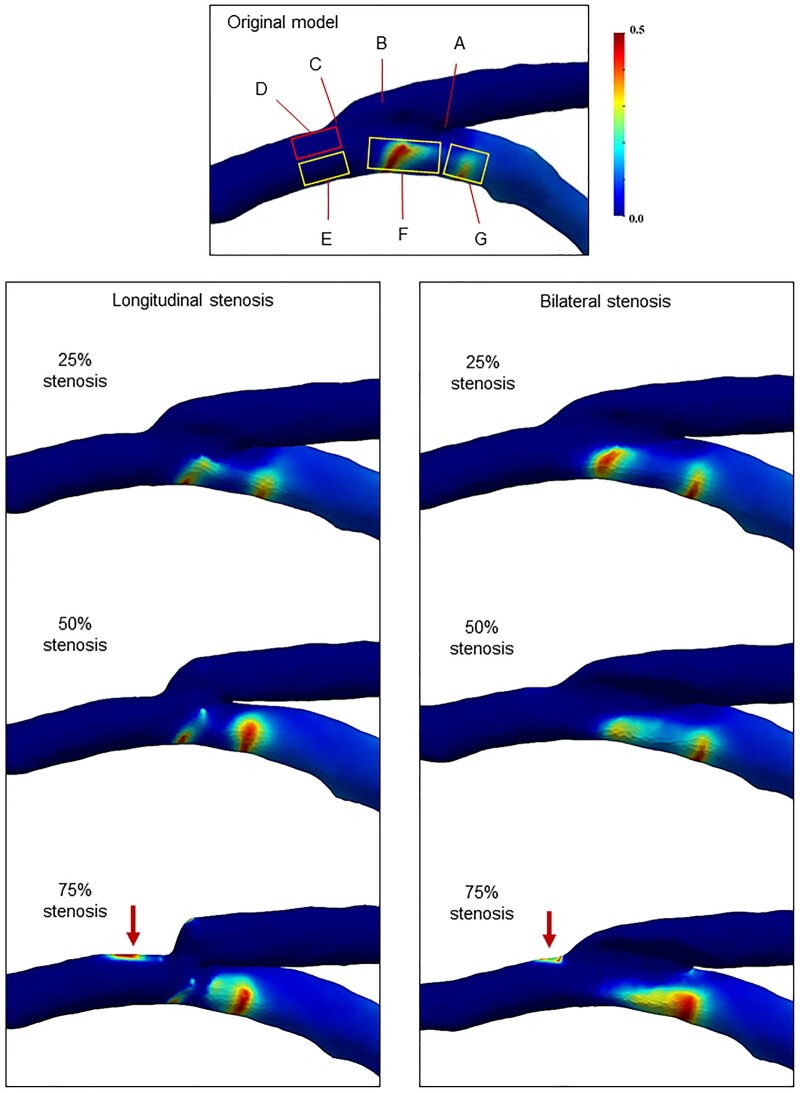
Oscillatory shear index (OSI) distributions of the end-to-side models with longitudinal or bilateral stenosis. In the original model, low-OSI areas were found at the heel, roof of the graft, toe and lower side of the distal toe (regions A, B, C and E). Conversely, high-OSI areas were located upstream of the heel (regions F and G), where the recirculation zone was located (Fig. 4). In 75% stenosis, high-OSI regions were identified at the upper side of the distal toe (red arrows, region D).

The distributions of WSS and OSI are shown in Figs 5 and 6, and Supplementary Material, Fig. S2, with the corresponding calculated values for the minimum WSS and maximum OSI in each region detailed in Supplementary Material, Tables S4–S7. In the original model, areas of low WSS were identified on the roof of the graft vessel (0.02 Pa, Fig. 5, region B) and at the floor of the heel within the native coronary artery (0.01 Pa, Fig. 5, region F). Conversely, high OSI areas were found beneath the heel and further proximally within the native coronary artery, where recirculation occurred (>0.44, Fig. 5, regions F and G).

In each stenosis model, the anastomosis site exhibited non-uniform distributions of large WSS gradients that intensified with increasing stenosis. In the case of longitudinal stenosis, the maximum WSS was predominantly concentrated at the toe (Fig. 5, region C), whereas the heel (Fig. 5, region A) showed high WSS values in the bilateral stenosis model. A comparison of each region in the longitudinal or bilateral stenosis models revealed that the superior side of the distal toe (Figs 5 and 6, region D), where flow separation occurred, exhibited a notable decrease in WSS (<0.05 Pa with 75% stenosis) and an increase in OSI (>0.46 with 75% stenosis) corresponding to the severity of the stenosis. Moreover, while the WSS at the inferior side of the distal toe (Figs 5 and 6, region E) showed a slight increase in the bilateral stenosis model (1.57 Pa with 75% stenosis), the OSI magnitudes in this region remained consistent across all stenosis models. To further visualize WSS and oscillatory motion on the vascular wall, the time-varying WSS is displayed in Supplementary Material, Video S3.

### The RRT in the native coronary artery

When comparing the effects of native coronary artery stenosis between 90% and 100%, the streamlines and distribution patterns of the WSS and OSI were almost identical across models with the same degree of stenosis (Supplementary Material, Figs S6–S8). However, for 100% native stenosis, the high RRT values were uniformly distributed on the proximal side of the recirculation region (Supplementary Material, Fig. S9). Conversely, with 90% native stenosis, areas of high RRT were scattered throughout the proximal native coronary artery (Supplementary Material, Fig. S4).

## DISCUSSION

Our study evaluated end-to-side anastomosis fluid dynamics, focusing on restrictive suturing, by simulating two stenotic patterns using an *ex vivo* porcine model. Both longitudinal and bilateral stenosis disrupted haemodynamic parameters, causing disturbed flow and abnormal focal changes in WSS and OSI. Notably, 75% stenosis in both patterns generated abnormal flow separation at the distal toe, creating low-WSS and high-oscillatory shear regions. Conversely, anastomotic stenoses of 25% and 50% induced only mild haemodynamic changes, potentially contributing to intimal hyperplasia. These findings suggest that mild to moderate stenosis (<50%) may pose an acceptable risk of intimal hyperplasia-associated graft failure following CABG.

Although some CFD-based studies have used patient-specific imaging data to construct CABG models for assessing haemodynamics, their simulation accuracy has been questioned due to the limited spatial resolution of CT, which may be insufficient for analysing small-diameter coronary arteries [14, 18]. Factors such as patients’ circulatory conditions, noise levels, data acquisition or reconstruction parameters and numerous artifacts can affect CFD performance. In our *ex vivo* models, ultra-high-resolution CT scanning precisely revealed anastomosis anatomy. Although clinical studies are required to connect simulations to outcomes, our method appears well-suited for simulating various anatomical variations and may provide haemodynamic insights into other CABG techniques, including side-to-side anastomosis, interrupted suturing, and sequential configurations.

The flow patterns and spatial WSS distribution in end-to-side anastomoses are complex. A distal end-to-side anastomosis induces abnormal flow due to its configuration and direction, unlike natural coronary bifurcations [9, 10]. *In vivo* studies have shown that intimal hyperplasia often develops at the toe, heel and arterial floor across the suture line, where flow disturbances and WSS gradients are common [10]. Our end-to-side model confirmed these findings, showing a recirculation zone (vortex flow) at the heel, flow impingement on the arterial floor and flow convergence in the distal region. Several histopathological and CFD-based studies have reported that such flow disturbances on the native arterial floor can damage the endothelium, potentially promoting atherogenesis and intimal hyperplasia [3, 9, 19]. The strong alignment between these findings and our simulation data supports the reliability of our CFD analysis.

Previous studies aimed to design optimal anastomosis configurations to enhance long-term graft patency [20, 21], showing that the anastomotic angle significantly affects haemodynamics, particularly the flow regime and shear stress [20]. Keynton *et al.* found that larger angles (30°, 45° and 60°) increased flow separation and disturbances around the toe, whereas smaller angles produced smoother flow [10, 20]. Our study showed effects similar to those of longitudinal stenosis, where a narrower anastomosis created a steeper flow angle. At 75% longitudinal stenosis, secondary flow separation emerged in the toe region, creating a highly oscillating zone.

Conversely, bilateral stenosis did not significantly distort the flow angle but generated helical flow along the lateral arterial walls with moderate to severe stenosis. Bilateral vortices likely form after passing through the stenosis, similar to normal vortical flow observed during early diastole beside the mitral leaflets in the left ventricle [22] (Supplementary Material, Fig. S3 and Supplementary Material, Video S2). In this study, 75% stenosis produced more extensive helical flow than 50% stenosis, due to concentrated inflow impacting a narrower arterial floor area and creating wider lateral spaces. This concentrated inflow also caused flow separation at the toe, similar to longitudinal stenosis. These results suggest that severe anastomotic stenosis, with increased resistance and narrowed inflow, may hinder bypass flow into the distal native coronary artery irrespective of the stenotic configuration patterns, potentially leading to graft failure.

Altered blood flow significantly impacts sheer stress on vascular endothelial cells [8]. In the CFD analysis, the distribution of high-WSS areas may indicate regions where local deformation of endothelial cells may increase vessel wall permeability, leading to intimal hyperplasia [3, 9]. In our study, the WSS gradient was relatively high along the anastomosis suture line in models with 75% stenosis, whereas the inferior side of the distal toe also showed high WSS in the bilateral 75% stenosis (Fig. 5). However, these WSS values may not be sufficiently high to directly cause intimal hyperplasia formation near the anastomosis. Research suggests the existence of a ‘safe bandwidth of WSS’, within which WSS levels are less likely to result in plaque formation [3, 9]: excessively high WSS (>38.9 Pa) may cause cell degeneration, whereas low WSS (<0.5 Pa), combined with high OSI, promotes intimal hyperplasia [11, 23]. Moreover, CFD analysis of a CABG model by Zhang *et al.* [24] indicated that regions characterized by low WSS and high OSI may be highly prone to atherosclerotic lesions. Based on these theories, the flow separation area at the distal toe, which provides a region of low WSS and high OSI in the 75% stenosis models, may pose a significant risk for graft failure. Furthermore, while 90% native coronary stenosis generated relatively slow recirculation flow, total occlusion (100% native stenosis) markedly increased RRT due to the absence of proximal native artery flow, potentially leading to platelet activation and thrombus formation, and subsequently contributing to intimal hyperplasia development.

### Limitations

This study had limitations. First, our computational analysis did not account for variable flow rates. As shown in Supplemental Material, Tables S2 and S3, flow rates in the graft and native coronary artery remained stable, likely due to partial compensation in our CFD model. For instance, when graft flow decreases through a stenotic lesion, native coronary flow tends to increase as compensation. Similar to other CFD-based studies, we used fixed boundary conditions from published measurements [13, 14]. The inlet aortic pressure and outlet peripheral resistance simulated flow through the graft and native coronary artery, although detailed intraluminal pressure measurements before and after anastomosis were unavailable, limiting the realism of the simulation. Although we focused on WSS and OSI as key predictive factors for long-term patency and atherosclerosis progression, a more detailed quantitative evaluation of blood flow variables in each region is needed.

Second, to manage computational costs, we simplified the multifactorial human cardiovascular system by treating vascular walls as rigid structures, disconnected from cardiac muscle contractions. Our simulation models lacked anatomical features, such as septal branches or collateral vessels, and did not simulate complex clinical conditions encompassing atherosclerosis, calcifications or heart failure [17]. Additionally, the velocity distribution, including streamlines, may be affected by several factors including the size and shapes of vascular models and the inlet and outlet boundary conditions. Thus, complex simulations encompassing coronary artery and graft variability could improve predictions of patient-specific outcomes.

Third, our end-to-side model only tested longitudinal shortening and bilateral narrowing patterns. In clinical practice, restrictive suturing in CABG can cause unifocal or unilateral stenosis [6, 7], which was not simulated in this study. Additionally, the model focused on grafting onto the LAD with an equal graft-to-host arteriotomy length. Future studies should incorporate various grafting patterns to calculate the optimal diameter ratios for different anastomotic configurations.

Finally, we did not validate the CFD simulation. Some experimental studies have used particle image velocimetry or other modalities for validation [9], although 4D flow magnetic resonance imaging analysis is limited by spatial and temporal resolution [16], making accuracy validation challenging. This CFD analysis methodology has been previously validated [16], but further data from direct measurements of actual coronary bypass flow are needed. Although we lack direct *in vivo* measurements for validation, our CFD analysis aligns with several previously reported *in vitro* studies [9].

## CONCLUSION

This CFD study, conducted using an *ex vivo* porcine cardiac model, enabled detailed haemodynamic simulations of end-to-side anastomoses with varying degrees of stenotic lesions. Compared to the original end-to-side model, longitudinal stenosis steepened the flow angle, whereas bilateral stenosis generated helical flow along the lateral wall. Both stenosis patterns caused flow separation in the distal toe region as the degree of stenosis increased. In cases of 75% severe anastomotic stenosis caused by longitudinal or lateral narrowing, regions of low WSS and high oscillatory shear were observed downstream of the anastomosis, whereas models with 25% and 50% stenosis showed little effect on these regions. These findings suggest that severely suboptimal configurations may induce abnormal flow and lead to irregular temporal and spatial strain distributions, potentially resulting in intimal hyperplasia within the vessel wall. Conversely, stenosis of less than 50% may be acceptable in local haemodynamics. Further clinical studies are warranted to validate these preliminary data and the long-term durability of an end-to-side anastomosis following CABG.

## Supplementary Material

ivaf013_Supplementary_Data

## Data Availability

The data underlying this article will be shared on reasonable request to the corresponding author.

## ABBREVIATIONS

CABG: Coronary artery bypass grafting
CFD: Computational fluid dynamics
CT: Computed tomography
LAD: Left anterior descending artery
OSI: Oscillatory shear index
RRT: Relative residence time
WSS: Wall shear stress
