# Supplementary material for: Computational fluid dynamics to simulate stenotic lesions in coronary end-to-side anastomosis
Source: Interdiscip Cardiovasc Thorac Surg. 2025 Jan 31;40(2):ivaf013. doi: 10.1093/icvts/ivaf013 (PMC11842071; doi:10.1093/icvts/ivaf013)
Supplement: ivaf013_Supplementary_Data [file ivaf013_supplementary_data.zip › CORRECT Supplementary Materials.docx]

SUPPLEMENTARY MATERIALS

**Computational fluid dynamics to simulate stenotic lesions in coronary end-to-side anastomosis**

Kenichi Kamiya, Shinya Terada, Yukihiro Nagatani, Yuji Matsubayashi, Kohei Suzuki, Shohei Miyazaki, Hiroki Matsui, Syota Takano, Susumu Nakata, Yoshiaki Watanabe, Tomoaki Suzuki

**TABLE OF CONTENTS**

**Supplementary methods for image data acquisition of the coronary artery bypass grafting model**

**Supplementary methods for image processing for surface segmentation**

**Supplementary methods for computational simulation**

**Supplementary Figure S1. Shape-blending technique to generate a stenotic end-to-side anastomosis model**

**Supplementary Figure S2. Antero-superior view of the streamlines, wall shear stress, and oscillatory shear index in the original model and with 75% of longitudinal and bilateral stenosis**

**Supplementary Figure S3. Anterior view of the streamlines in the end-to-side models with longitudinal and bilateral stenosis**

**Supplementary Figure S4. The RRT distribution in the proximal native coronary artery with longitudinal and bilateral stenosis and 90% native coronary stenosis**

**Supplementary Figure S5. Boundary conditions of the CFD analysis with 100% native coronary stenosis**

**Supplementary Figure S6. Streamlines in end-to-side anastomosis, with longitudinal or bilateral stenosis and 100% native coronary stenosis**

**Supplementary Figure S7. The WSS distributions in the end-to-side anastomosis with longitudinal or bilateral stenosis and 100% native coronary stenosis**

**Supplementary Figure S8. The OSI distributions in the end-to-side models with longitudinal or bilateral stenosis and 100% native coronary stenosis**

**Supplementary Figure S9. The RRT distribution in the proximal native coronary artery with longitudinal or bilateral stenosis patterns and 100% native coronary stenosis**

**Supplementary Table S1. Geometry of the end-to-side anastomosis in the original and created stenotic models**

**Supplementary Table S2. Flow rates through the end-to-side anastomosis with 90% coronary artery stenosis**

**Supplementary Table S3. Flow rates through the end-to-side anastomosis with 100% coronary artery stenosis**

**Supplementary Table S4. Minimum values of WSS in the end-to-side anastomosis with 90% native coronary artery stenosis**

**Supplementary Table S5. Maximum values of OSI in the end-to-side anastomosis with 90% native coronary artery stenosis**

**Supplementary Table S6. Minimum values in WSS of the end-to-side anastomosis with 100% native coronary artery stenosis**

**Supplementary Table S7. Maximum values of OSI in the end-to-side anastomosis with 100% native coronary artery stenosis**

**Supplementary methods for image data acquisition of the coronary artery bypass grafting model**

The grafted porcine heart was positioned with the anastomosis facing upward for computed tomography (CT) scanning. Image data were acquired from the silicone cast model using a 160-row multidetector CT scanner (Aquilion Precision, Canon Medical Systems, Otawara, Tochigi, Japan) in ultra-high-resolution mode. The data acquisition and reconstruction parameters were as follows: tube voltage = 120 kVp, tube current-time product = 75 mAs, reconstruction slice thickness and increments = 0.25 mm, reconstruction field of view = 170 mm, and reconstruction kernel = FC04 (standard).

**Supplementary methods for image processing for surface segmentation**

Image data in Digital Imaging and Communications in Medicine format were transferred into Vesalius3D software (version 2.12; PS-tech, Amsterdam, Netherlands). Using the segmentation tools within Vesalius3D, the mask volume of the end-to-side anastomosis was semi-automatically extracted (Figure 1). The segmented volume masks were then saved as standard tessellation language files in the highest-resolution format.

**Supplementary methods for computational simulation**

*Boundary conditions*

In the computational fluid dynamics (CFD) analysis, the inlet and outlet flow conditions were carefully defined at the boundaries of the simulation domain. The velocity and pressure variables of the blood flow within this domain were calculated using the CFD analysis, adhering to both the continuity and Navier–Stokes equations. To simulate flow in the anastomosed region, a simulation model was developed that allows for the explicit specification of time-varying flow rates and pressures as boundary conditions, with subsequent calculations of the flow rates in the graft and native coronary artery (Figure 3). Consequently, stable numerical solutions were calculated for a range of end-to-side anastomosis variations. In this model, the inlet boundary condition at the aortic root was set to a flow rate of 5.0 L/min with a pulsatile waveform, whereas the outlet boundary condition at the ascending aorta was maintained at an average pressure of 96 mmHg (13, 14). Time-varying impedance boundary conditions, reflecting physiological peripheral resistance during one cardiac cycle, were applied to the coronary artery outlet boundaries in the LAD to simulate peripheral vascular beds. This peripheral coronary impedance was estimated based on variable pulse and flow wave patterns to represent myocardial perfusion volume for distal branches during ventricular muscle contraction and relaxation. These profiles were adopted from previously published data based on direct pressure and velocity measurements at the aorta and distal coronary artery branches (14). The vessel walls, including the extended boundary walls, were modeled as rigid structures.

*Pulsatile flow analysis*

The finite volume method was employed to solve the mass and momentum conservation equations (Navier–Stokes equation) and the continuity equation using the open-source CFD software program OpenFOAM version 8.0 (OpenFOAM Foundation, London, UK). Blood was treated as an incompressible Newtonian fluid with a density of 1060 kg/m^3^ and a viscosity of 0.004 Pa·s. Each simulation was run for one cardiac cycle (1 sec) to obtain a periodic solution representing physiologic pulsatile flow. For transient flow analysis, each time step was set to 10^-5^ sec to achieve a sufficiently low Courant number. In the iterative process of solving the matrix solutions, the convergence criteria were set to 10^-5^ times the residual for all degrees of the parameters. Applying a Reynolds number of approximately 400, we assumed a laminar flow scheme without turbulence through the LAD and graft, except at the anastomotic site in our models. Additionally, we calculated sufficiently low energy loss in native coronary and graft vessels, excluding the anastomotic region. The calculated results that achieved convergence were postprocessed to visualize and quantify the hemodynamic parameters using ParaView 5.1.1 (Kitware, Inc., New York, USA). Detailed computations and analyses were conducted by Cardio Flow Design Inc., Japan (13-15). The CFD methodology was based on that of prior validated studies (16).

*Anastomosis evaluations of near-wall hemodynamics*

From the computational results, we evaluated the visualized blood flow patterns (“streamlines”) and analyzed velocity vectors. Near-wall hemodynamics were calculated, including wall shear stress (WSS), its fluctuations as the oscillatory shear index (OSI) (15), and the relative residence time (RRT) (17). While these parameters were transiently analyzed during the diastolic phase (at 0.7 sec), exhibiting a peak flow rate in the native coronary artery, the fluctuations in streamlines, velocity vectors, and WSS were visualized in one cardiac cycle. The WSS, which is the tangential frictional force exerted on the vessel’s inner surface, was determined based on the pulsatile flow and the complex geometric structures of the vessels. For a Newtonian fluid, shear stress is directly proportional to the flow shear rate (change in flow velocity across a vessel) and the dynamic blood viscosity, μ. This relationship for a straight vessel can be expressed by the following formula:

$$\vec{WSS}=\mu\frac{\partial\vec{u}}{\partial y}$$

The OSI quantifies the changes in both the direction and magnitude of the WSS and is calculated using the following formula:

$$OSI= \frac{1}{2}(1-\frac{\left\| \int_{0}^{T} \vec{WSS}dt \right\|}{\int_{0}^{T} \left\| \vec{WSS} \right\|dt})$$

Here, the numerator represents the total WSS (the sum of all positive and negative WSS vectors) and the denominator reflects the sum of all absolute values of these WSS vectors over one cardiac cycle. The OSI values range from 0 to 0.5, with 0 indicating purely unidirectional WSS and 0.5 representing a fully multidirectional, oscillatory flow. High OSI values are associated with endothelial dysfunction and plaque progression (3).

Additionally, the RRT is a metric used to identify regions of disturbed and adverse blood flow that may increase the risk of atherosclerosis or thrombotic stenosis (17). The RRT is proportional to the magnitude of the time-averaged wall shear stress (TAWSS), calculated using the following equations:

$$TAWSS= \frac{1}{T}\left\| \int_{0}^{T} \vec{WSS}dt \right\|$$

$$RRT= \frac{1}{\left( 1-2*OSI \right)*TAWSS}$$

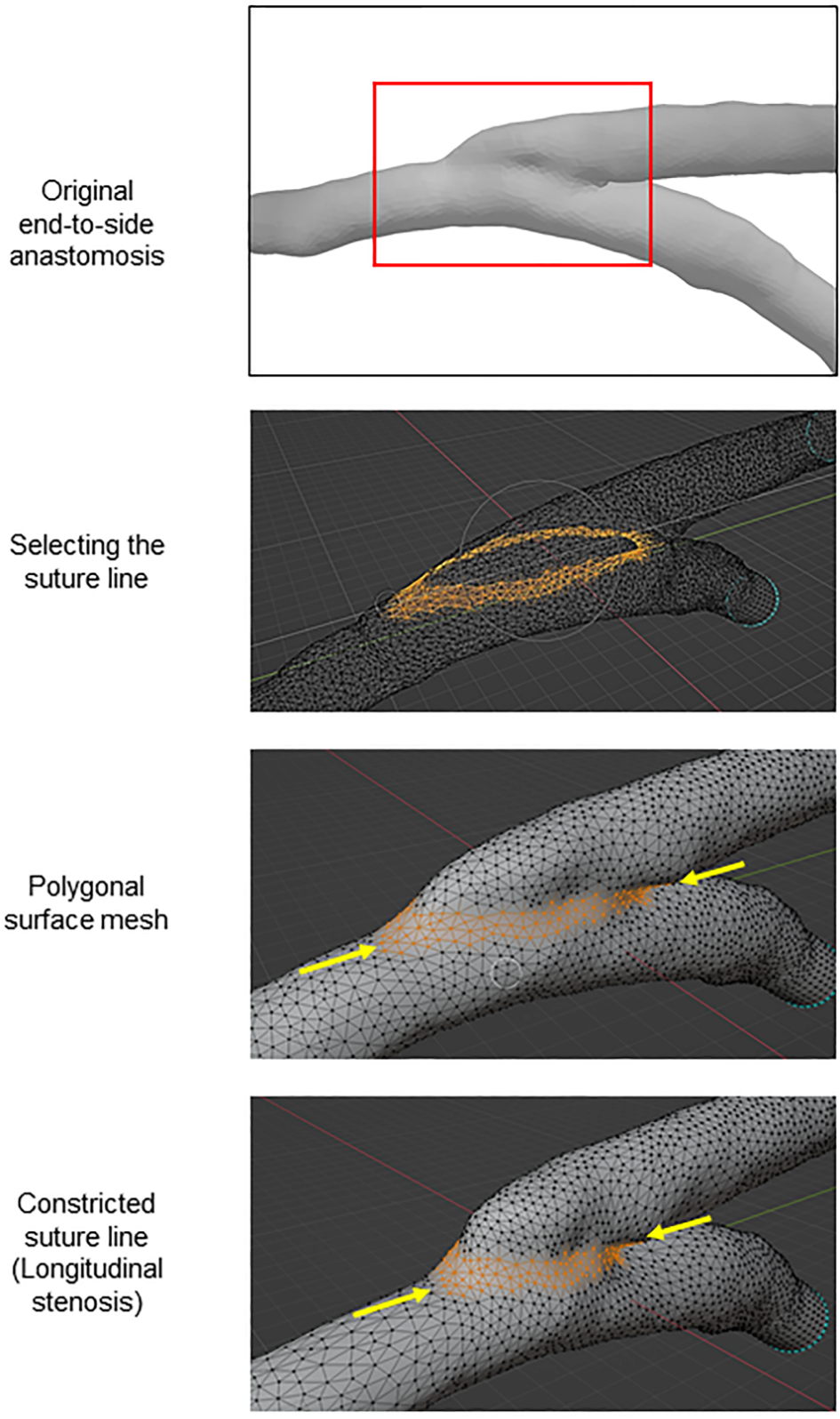


**Supplementary Figure S1. Shape-blending technique to generate a stenotic end-to-side anastomosis model**

The anastomotic suture line of the original end-to-side model (red box) was specified to identify the heel and toe edges as well as the lateral borders (orange colored area). When generating longitudinal stenosis, both edges (heel and toe) were transformed toward the center of the anastomosis (yellow arrows).


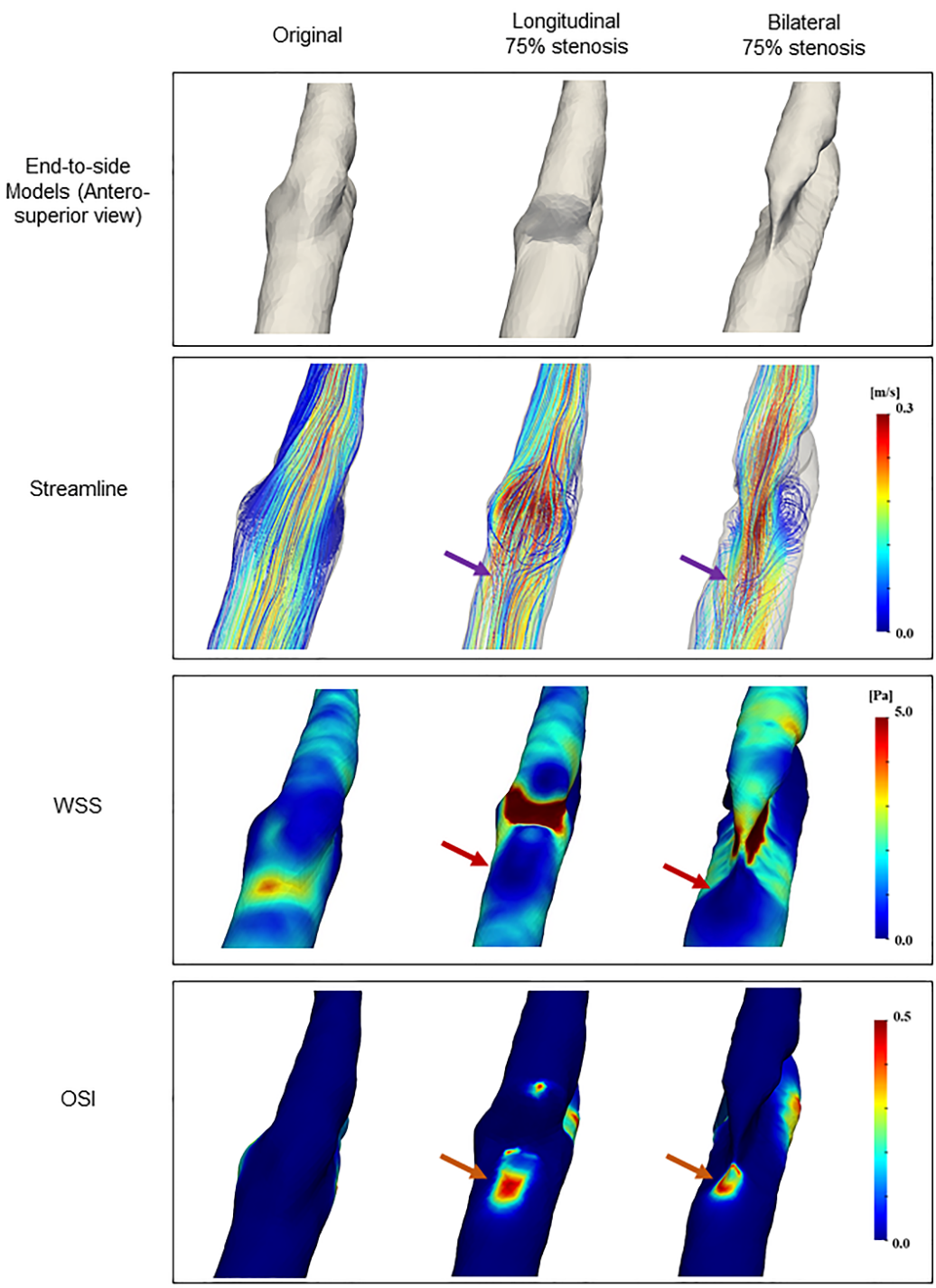


**Supplementary Figure S2. Antero-superior view of the streamlines, wall shear stress, and oscillatory shear index in the original model, and with 75% of longitudinal and bilateral stenosis**

With 75% stenosis, both stenotic patterns involved a reversed-flow region due to flow separations downstream of the anastomosis (purple arrows). There were also low WSS areas in the region distal to the toe of the native coronary artery (red arrows). Highly oscillating regions were found at the distal toe region (orange arrows), corresponding to the stagnation point. (WSS, wall shear stress; OSI, oscillating shear index).


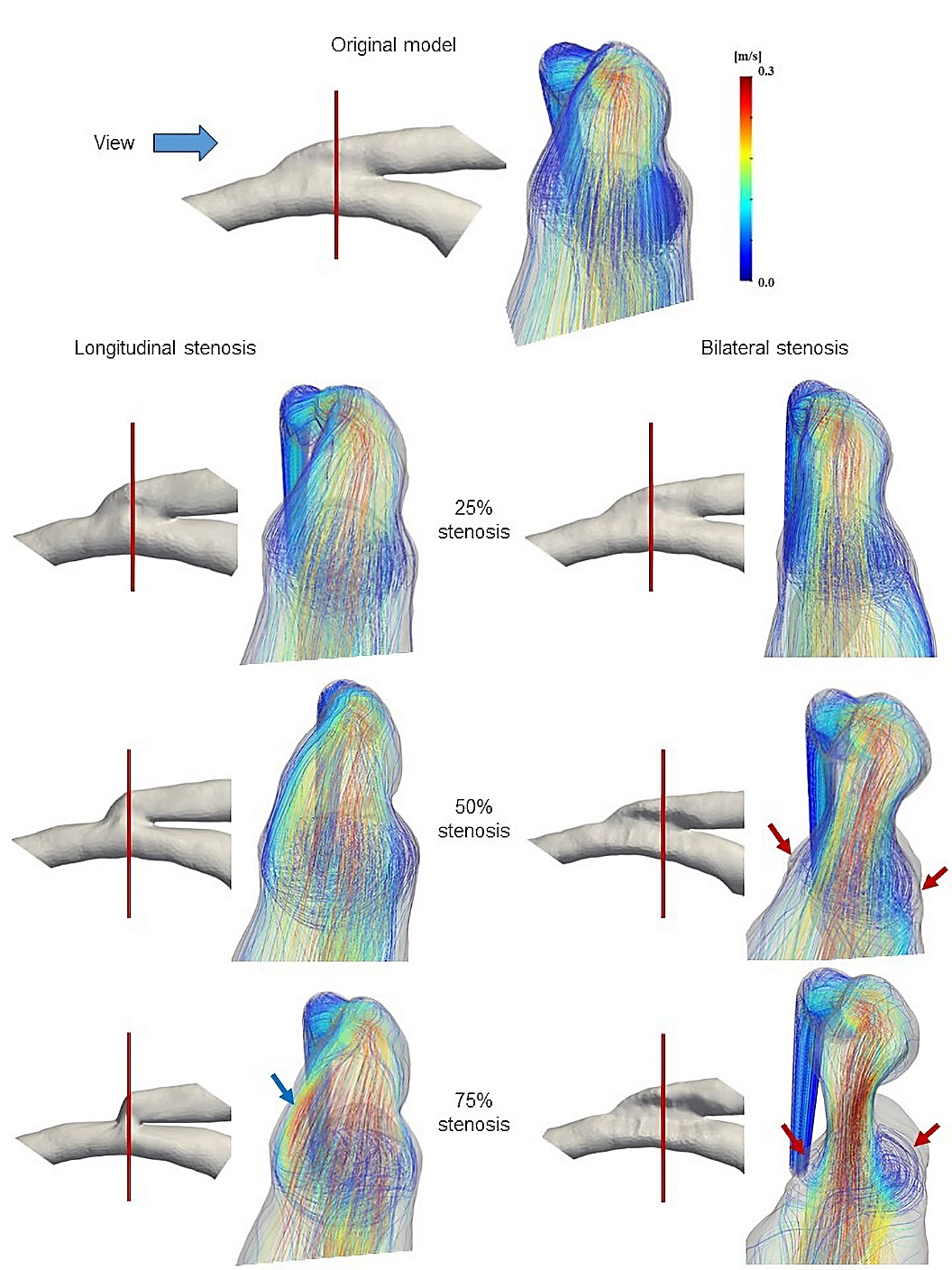


**Supplementary Figure S3. Anterior view of the streamlines in the end-to-side models with longitudinal and bilateral stenosis**

Streamlines representing the anterior view orthogonal to the perpendicular line of the anastomosis (red line). With longitudinal stenosis, the graft inflow through the narrowed anastomotic region shows increased velocity toward the floor of the native coronary artery (blue arrow). Conversely, with bilateral stenosis, narrowed inflow through the stenotic region directed toward the floor of the native coronary artery, spreading laterally to generate bilateral vortices beside the central inflow (red arrows).


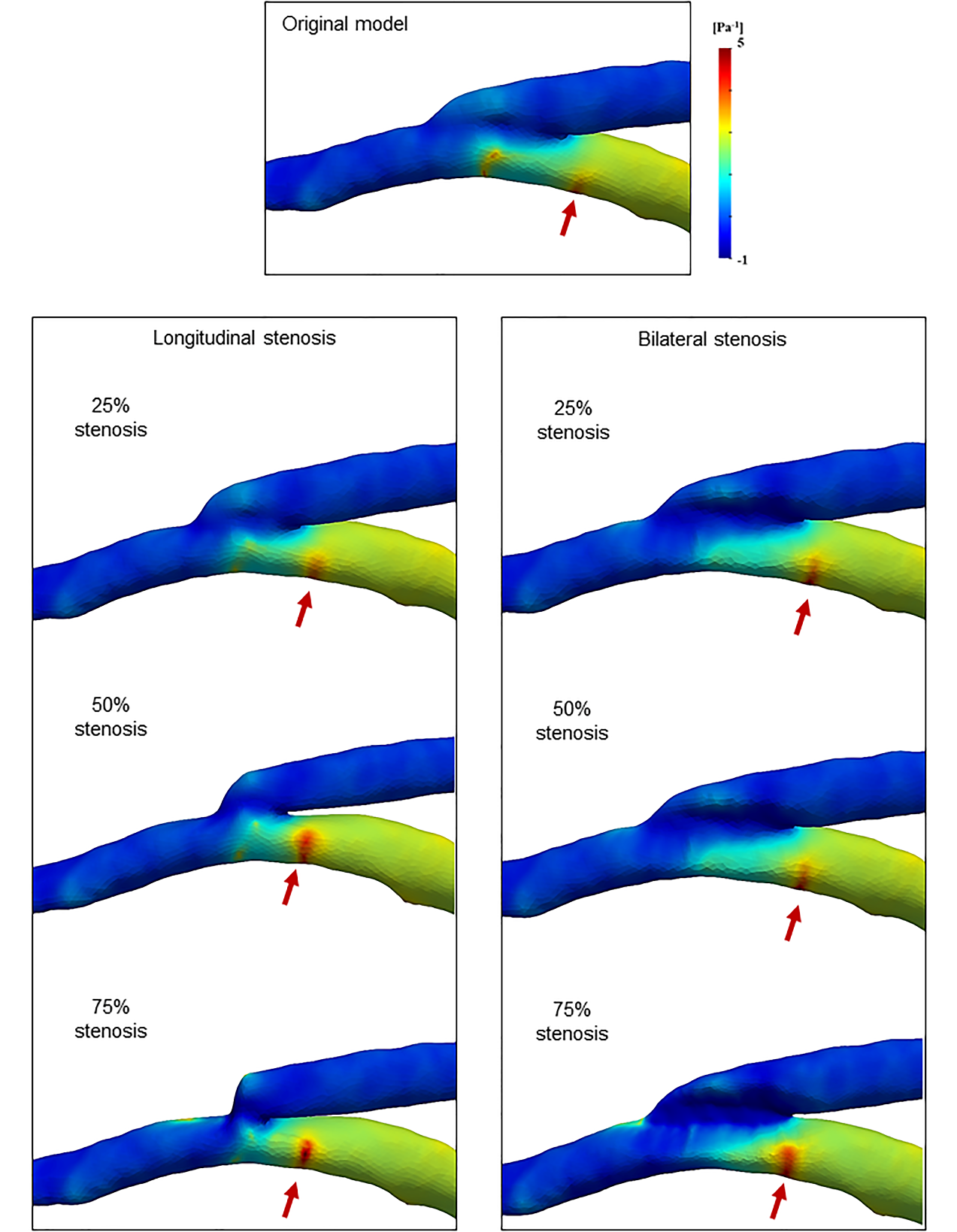


**Supplementary Figure S4. The RRT distribution in the proximal native coronary artery with longitudinal and bilateral stenosis and 90% native coronary stenosis**

With 90% coronary stenosis, there are scattered high-RRT areas distributed at the proximal side of the native coronary artery, corresponding to the recirculation region (red arrows) (RRT: relative residence time).

**
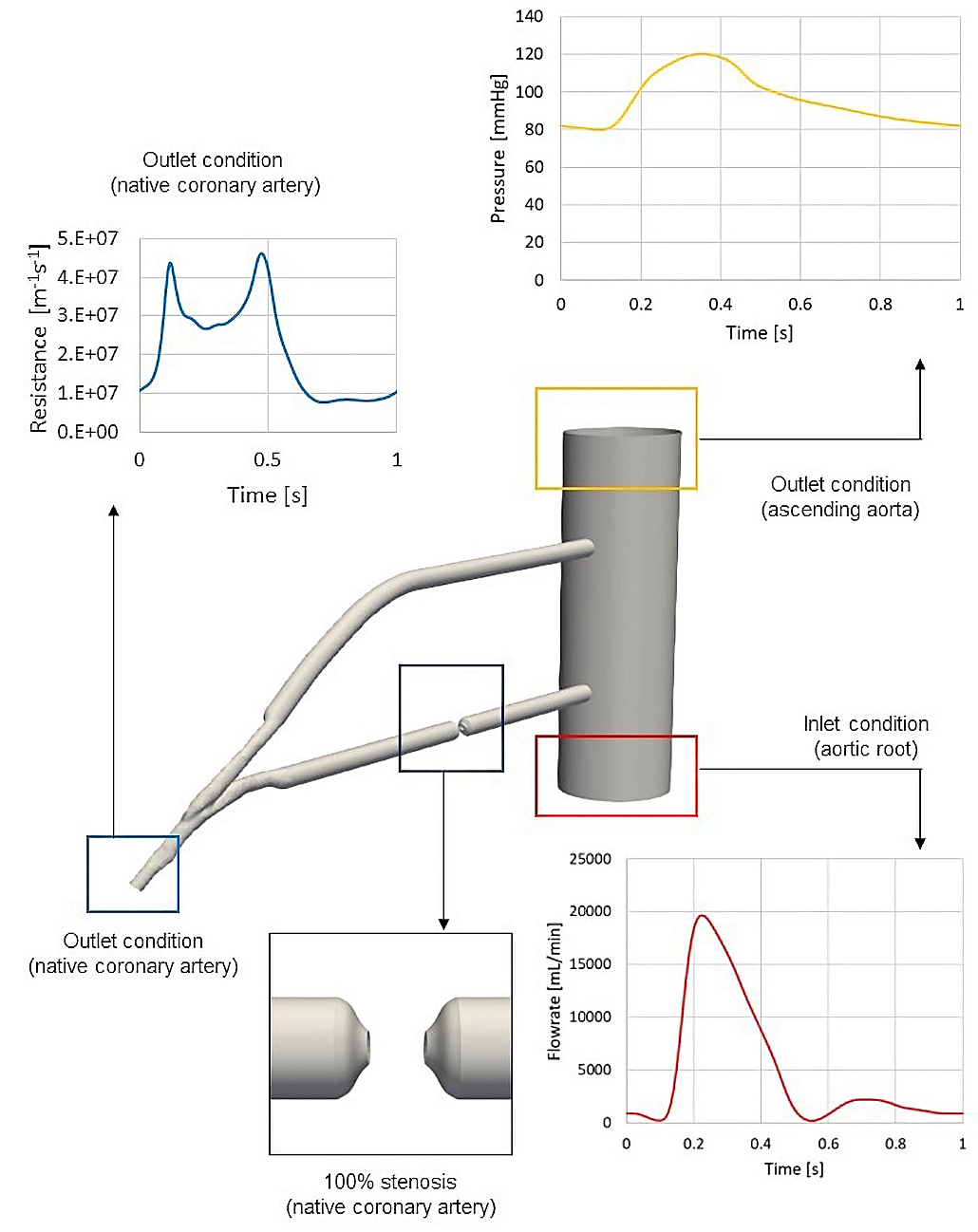
**

**Supplementary Figure S5. Boundary conditions of the CFD analysis with 100% native coronary stenosis**

To simulate the flow in the anastomosed region, the inlet flow rate of the aortic root was set to 5.0 L/min with a pulsatile wave (red box) and the outlet pressure of the ascending aorta was set to an average of 96 mmHg (yellow box). Time-varying impedance boundary conditions were used for the coronary artery outlet boundary conditions (blue box) (CFD: computational fluid dynamics).


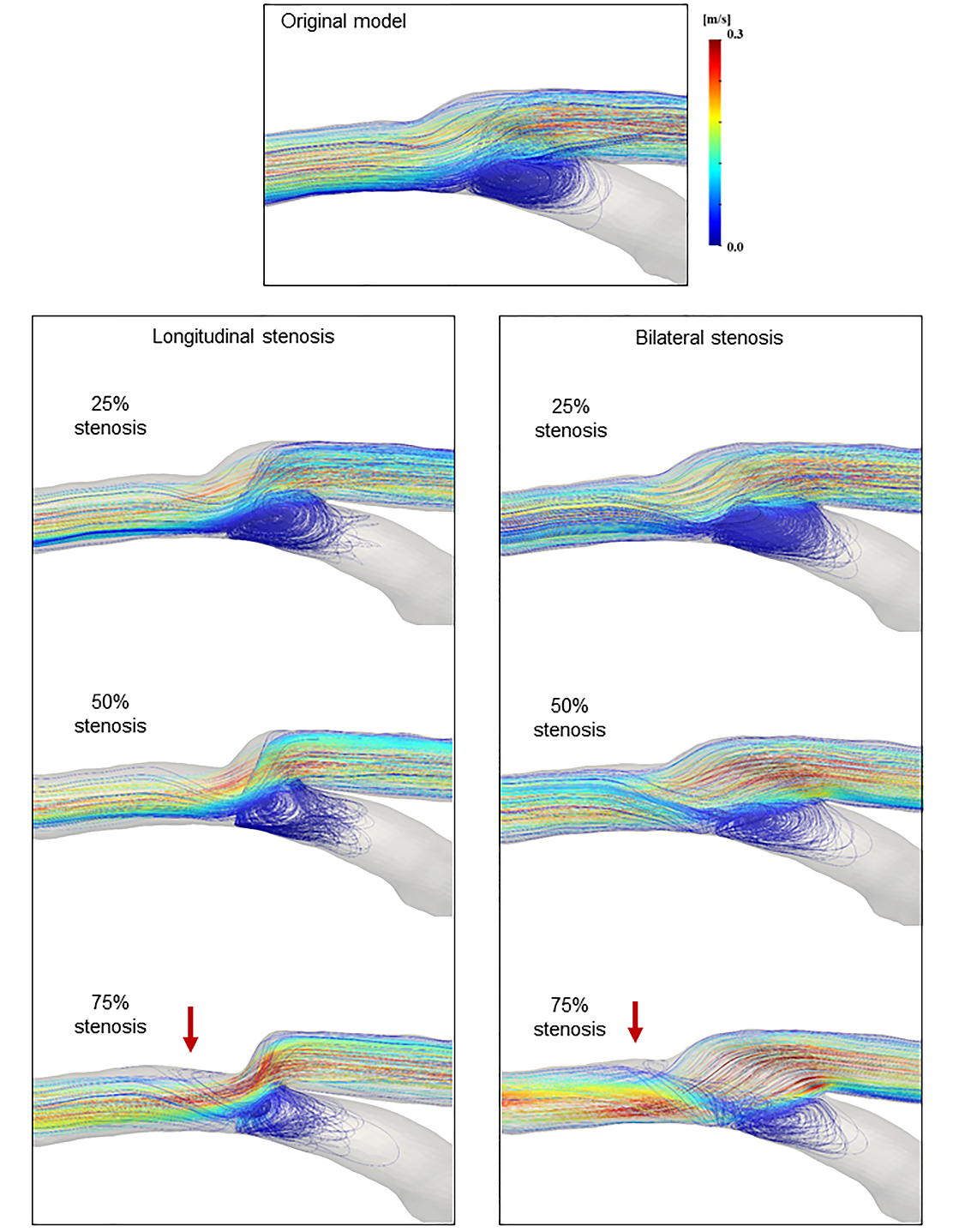


**Supplementary Figure S6. Streamlines in the end-to-side with longitudinal or bilateral stenosis with 100% native coronary stenosis**

A typical flow pattern in the original end-to-side model is characterized by a recirculation zone (vortex flow) in the heel region, flow impinging on the artery floor, and flow gathering in the distal region. With longitudinal stenosis, the narrower anastomosis resulted in a steeper flow angle. In contrast, with bilateral stenosis, the spiral motion of helical flow was generated along the lateral arterial wall. With 75% stenosis of either shape, the flow impinged on the native artery floor, creating flow separation at the toe (red arrows).


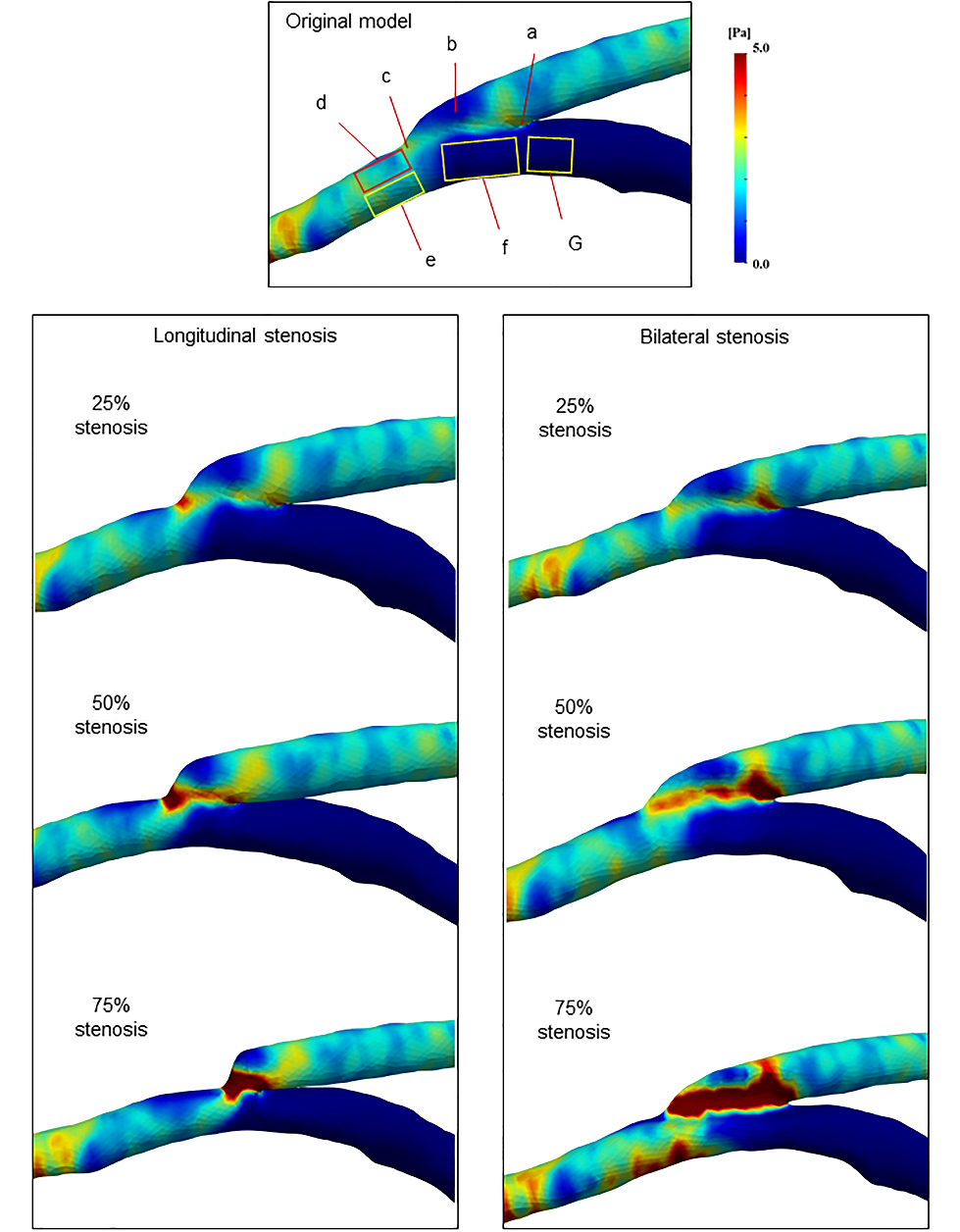


**Supplementary Figure S7. The WSS distributions in the end-to-side anastomosis with longitudinal or bilateral stenosis, and 100% native coronary stenosis.**

In the original model, there were relatively high WSS areas at the heel (region A) and toe (region C), whereas low WSS areas were found at the roof of the graft (region B), floor of the heel (region F), and upstream of the native coronary artery (region G). With longitudinal stenosis, the maximum WSS was generated at the toe, whereas high WSS lesion was predominantly found at the heel with bilateral stenosis. With 75% stenosis, both longitudinal and bilateral stenosis models show a low WSS area at the upper side of the distal toe (region D), whereas the lower side of the distal toe (region E) shows relatively higher WSS with bilateral stenosis (WSS: wall shear stress).


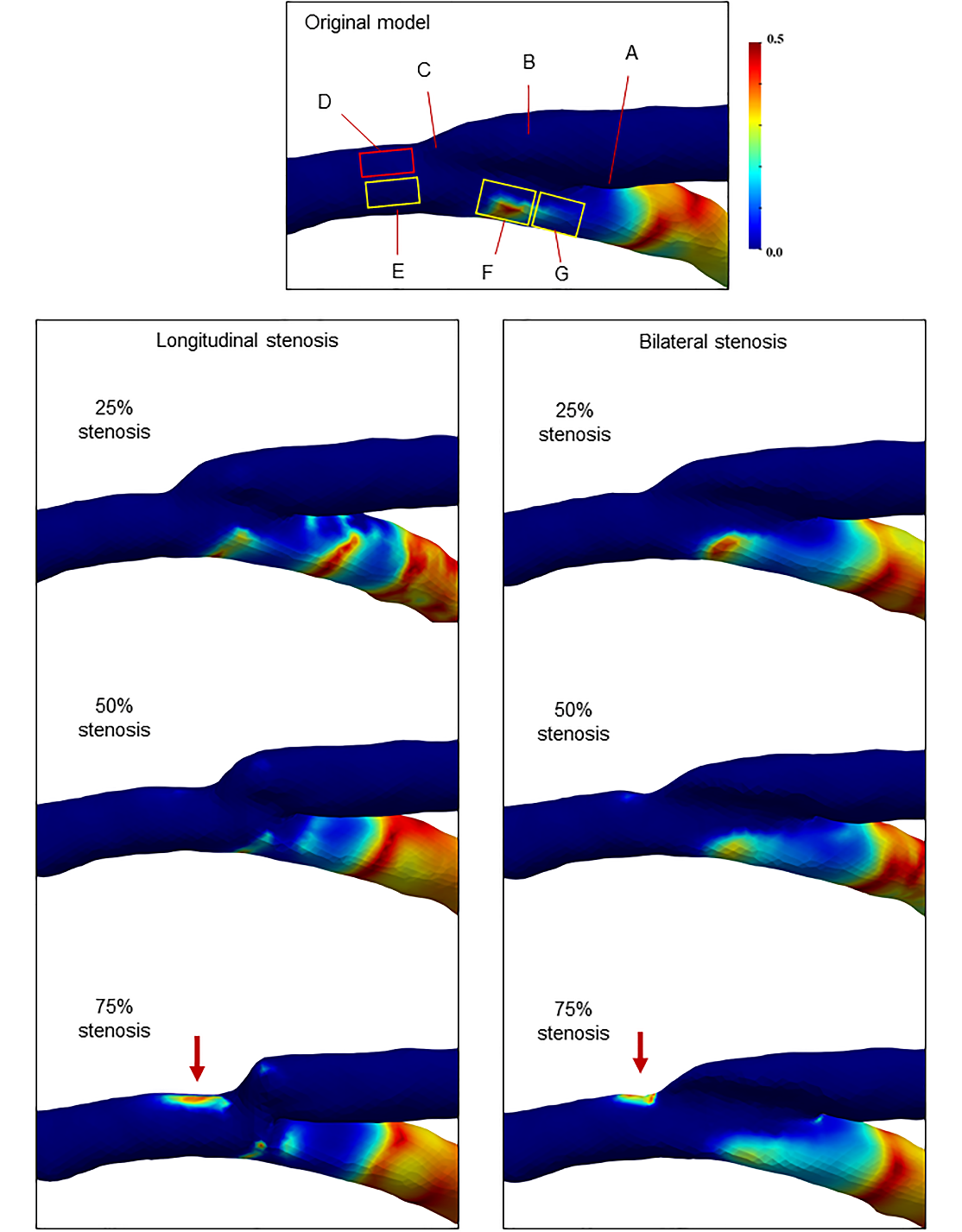


**Supplementary Figure S8. The OSI distributions in the end-to-side models with longitudinal or bilateral stenosis patterns and 100% native coronary stenosis**

In the original model, the low-OSI areas were found at the heel (region A), roof of the graft (region B), toe (region C), and lower side of the distal toe (region E). Conversely, the high-OSI areas were found upstream in the heel (regions F and G), where the recirculation zone was located (Figure 4). In the 75% stenosis models, highly oscillating regions were found at the upper side of the distal toe (region D), corresponding to the flow stagnation points (Figure 4) (OSI: oscillatory shear index).


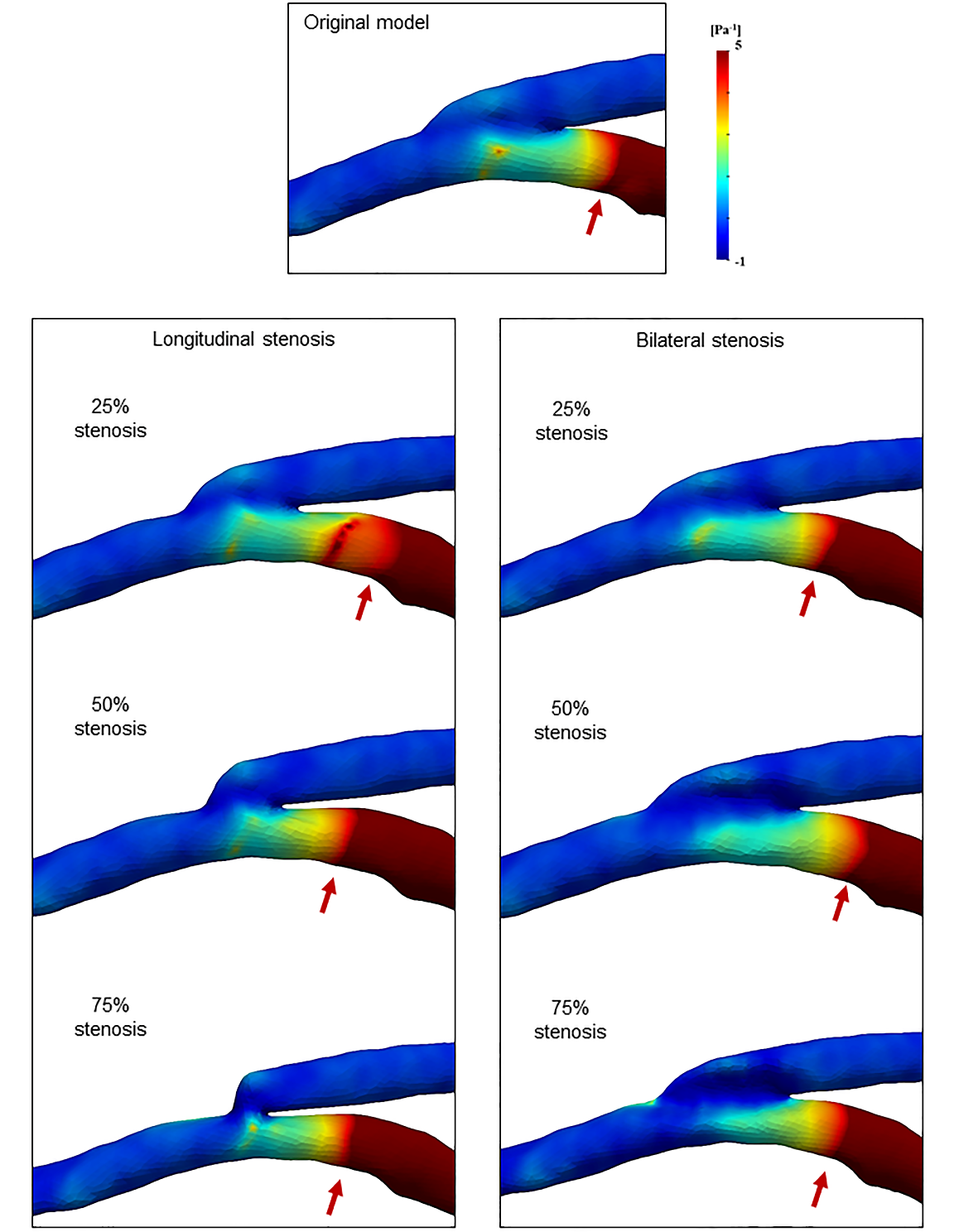


**Supplementary Figure S9. The RRT distribution in the proximal native coronary artery with longitudinal or bilateral stenosis patterns and 100% native coronary stenosis**

With 100% coronary stenosis, there are high-RRT areas uniformly distributed at the proximal side of the native coronary artery, corresponding to the recirculation region (red arrows) (RRT: relative residence time).

**Supplementary Table S1. Geometry of the end-to-side anastomosis in the original and created stenotic models**

|  | **Longitudinal diameter (mm)** | **Transverse diameter (mm)** | **Perimeter (mm)** | **Area (mm^2^)** |
| --- | --- | --- | --- | --- |
| **Original model** | 9.1 | 4.4 | 21.5 | 28.7 |
| **Longitudinal stenosis** |  |  |  |  |
| 25% | 6.8 | 4.4 | 17.5 | 21.5 |
| 50% | 4.5 | 4.4 | 13.7 | 14.4 |
| 75% | 2.3 | 4.4 | 10.5 | 7.2 |
| **Transverse stenosis** |  |  |  |  |
| 25% | 9.1 | 3.3 | 20.3 | 21.5 |
| 50% | 9.1 | 2.2 | 19.4 | 14.3 |
| 75% | 9.1 | 1.1 | 18.7 | 7.1 |

**Supplementary Table S2. Flow rates through the end-to-side anastomosis with 90% coronary artery stenosis**

|  | **Graft**  **(ml/min)** | **Distal coronary artery**  **(ml/min)** | **Proximal coronary artery (ml/min)** |
| --- | --- | --- | --- |
| **Original model** | 44.2 | 44.7 | 0.294 |
| **Longitudinal stenosis** |  |  |  |
| 25% | 43.7 | 44.7 | 0.283 |
| 50% | 44.0 | 44.1 | 0.319 |
| 75% | 43.7 | 44.1 | 0.356 |
| **Bilateral stenosis** |  |  |  |
| 25% | 43.8 | 44.3 | 0.298 |
| 50% | 43.9 | 44.7 | 0.324 |
| 75% | 45.3 | 45.0 | 0.416 |

**Supplementary Table S3. Flow rates through the end-to-side anastomosis with 100% coronary artery stenosis**

|  | **Graft**  **(ml/min)** | **Distal coronary artery**  **(ml/min)** | **Proximal coronary artery (ml/min)** |
| --- | --- | --- | --- |
| **Original model** | 43.9 | 43.9 | - |
| **Longitudinal stenosis** |  |  |  |
| 25% | 45.3 | 45.3 | - |
| 50% | 45.0 | 45.0 | - |
| 75% | 45.0 | 45.0 | - |
| **Bilateral stenosis** |  |  |  |
| 25% | 44.3 | 44.3 | - |
| 50% | 43.9 | 43.9 | - |
| 75% | 45.4 | 45.4 | - |

**Supplementary Table S4. Minimum values of WSS in the end-to-side anastomosis with 90% native coronary artery stenosis**

|  | **Region*** | | | | | | |
| --- | --- | --- | --- | --- | --- | --- | --- |
|  | **Heel** | **Roof of the graft** | **Toe** | **Upper side of the distal toe** | **Lower side of the distal toe** | **Floor of the heel** | **Upstream of the heel** |
| **Original model** | 2.59 | 0.41 | 3.00 | 1.03 | 1.33 | 0.01 | 0 |
| **Longitudinal stenosis** |  |  |  |  |  |  |  |
| 25% | 3.93 | 0.45 | 4.81 | 0.80 | 1.43 | 0.01 | 0 |
| 50% | 4.17 | 0.57 | 7.75 | 0.35 | 1.11 | 0.01 | 0 |
| 75% | 7.71 | 0.82 | 8.31 | 0.05 | 0.60 | 0.01 | 0 |
| **Bilateral stenosis** |  |  |  |  |  |  |  |
| 25% | 4.53 | 0.54 | 3.36 | 0.74 | 1.06 | 0.01 | 0 |
| 50% | 6.98 | 0.75 | 3.71 | 0.29 | 1.29 | 0.01 | 0 |
| 75% | 11.3 | 0.94 | 6.13 | 0.02 | 1.57 | 0.01 | 0 |

*Locations of regions are shown in Figure 5.

WSS: wall shear strength

**Supplementary Table S5. Maximum values of OSI in the end-to-side anastomosis with 90% native coronary artery stenosis**

|  | **Region*** | | | | | | |
| --- | --- | --- | --- | --- | --- | --- | --- |
|  | **Heel** | **Roof of the graft** | **Toe** | **Upper side of the distal toe** | **Lower side of the distal toe** | **Floor of the heel** | **Upstream of the heel** |
| **Original model** | 0 | 0 | 0 | 0 | 0.04 | 0.46 | 0.44 |
| **Longitudinal stenosis** |  |  |  |  |  |  |  |
| 25% | 0 | 0 | 0 | 0 | 0.03 | 0.41 | 0.45 |
| 50% | 0 | 0 | 0 | 0 | 0.03 | 0.36 | 0.41 |
| 75% | 0 | 0 | 0 | 0.46 | 0.03 | 0.40 | 0.48 |
| **Bilateral stenosis** |  |  |  |  |  |  |  |
| 25% | 0 | 0 | 0 | 0.01 | 0.02 | 0.25 | 0.36 |
| 50% | 0 | 0 | 0 | 0.04 | 0 | 0.19 | 0.43 |
| 75% | 0 | 0 | 0 | 0.46 | 0 | 0.21 | 0.47 |

*Locations of regions are shown in Figure 6.

OSI: oscillatory shear index

**Supplementary Table S6. Minimum values of WSS in the end-to-side anastomosis with 100% native coronary artery stenosis**

|  | **Region*** | | | | | | |
| --- | --- | --- | --- | --- | --- | --- | --- |
|  | **Heel** | **Roof of the graft** | **Toe** | **Upper side of the distal toe** | **Lower side of the distal toe** | **Floor of the heel** | **Upstream of the heel** |
| **Original model** | 2.81 | 0.45 | 2.93 | 1.10 | 1.38 | 0.01 | 0 |
| **Longitudinal stenosis** |  |  |  |  |  |  |  |
| 25% | 3.69 | 0.52 | 4.47 | 0.71 | 0.30 | 0.01 | 0 |
| 50% | 4.80 | 0.67 | 7.78 | 0.21 | 0.62 | 0.01 | 0.01 |
| 75% | 9.75 | 0.93 | 8.44 | 0.01 | 0.31 | 0.02 | 0 |
| **Bilateral stenosis** |  |  |  |  |  |  |  |
| 25% | 4.46 | 0.56 | 3.22 | 0.71 | 1.38 | 0.01 | 0.01 |
| 50% | 6.46 | 0.66 | 3.37 | 0.22 | 1.13 | 0.01 | 0.02 |
| 75% | 13.9 | 0.86 | 8.25 | 0.04 | 1.44 | 0 | 0.02 |

*Locations of regions are shown in Supplementary Figure S6.

WSS: wall shear strength

**Supplementary Table S7. Maximum values of OSI in the end-to-side anastomosis with 100% native coronary artery stenosis**

|  | **Region*** | | | | | | |
| --- | --- | --- | --- | --- | --- | --- | --- |
|  | **Heel** | **Roof of the graft** | **Toe** | **Upper side of the distal toe** | **Lower side of the distal toe** | **Floor of the heel** | **Upstream of the heel** |
| **Original model** | 0 | 0 | 0 | 0 | 0.04 | 0.46 | 0.44 |
| **Longitudinal stenosis** |  |  |  |  |  |  |  |
| 25% | 0 | 0 | 0 | 0.01 | 0.12 | 0.38 | 0.41 |
| 50% | 0 | 0 | 0 | 0.05 | 0.06 | 0.42 | 0.06 |
| 75% | 0 | 0 | 0 | 0.36 | 0.06 | 0.41 | 0.03 |
| **Bilateral stenosis** |  |  |  |  |  |  |  |
| 25% | 0 | 0 | 0 | 0.01 | 0 | 0.27 | 0.37 |
| 50% | 0 | 0 | 0 | 0.09 | 0 | 0.21 | 0.13 |
| 75% | 0 | 0 | 0 | 0.45 | 0 | 0.22 | 0.15 |

*Locations of regions are shown in Supplementary Figure S7.

OSI: oscillatory shear index
