# Supplementary figures and images for: Computational fluid dynamics to simulate stenotic lesions in coronary end-to-side anastomosis
Source: Interdiscip Cardiovasc Thorac Surg. 2025 Jan 31;40(2):ivaf013. doi: 10.1093/icvts/ivaf013 (PMC11842071; doi:10.1093/icvts/ivaf013)

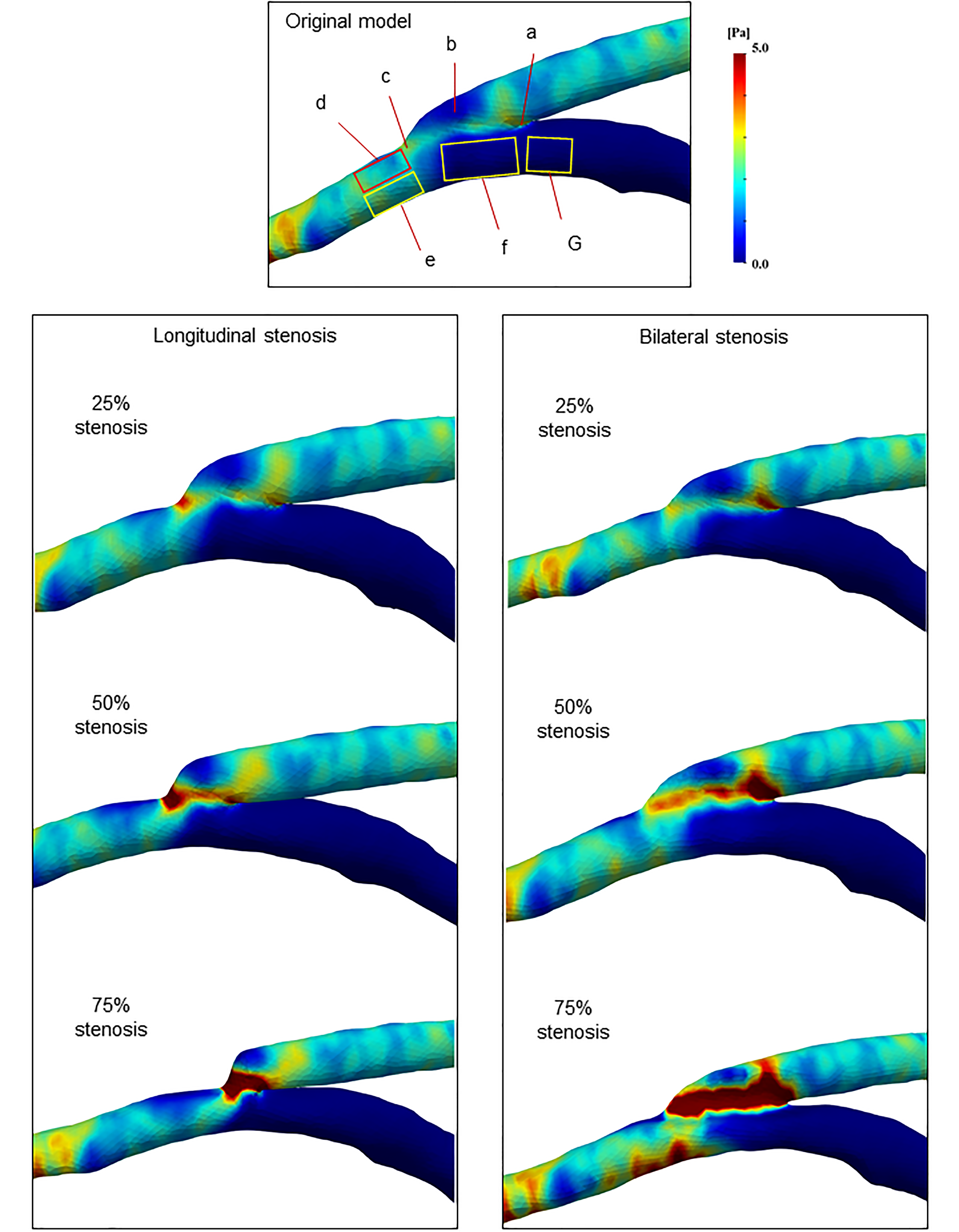

Supplement: ivaf013_Supplementary_Data [file ivaf013_supplementary_data.zip › Figure_S7__2nd_version_.TIF]

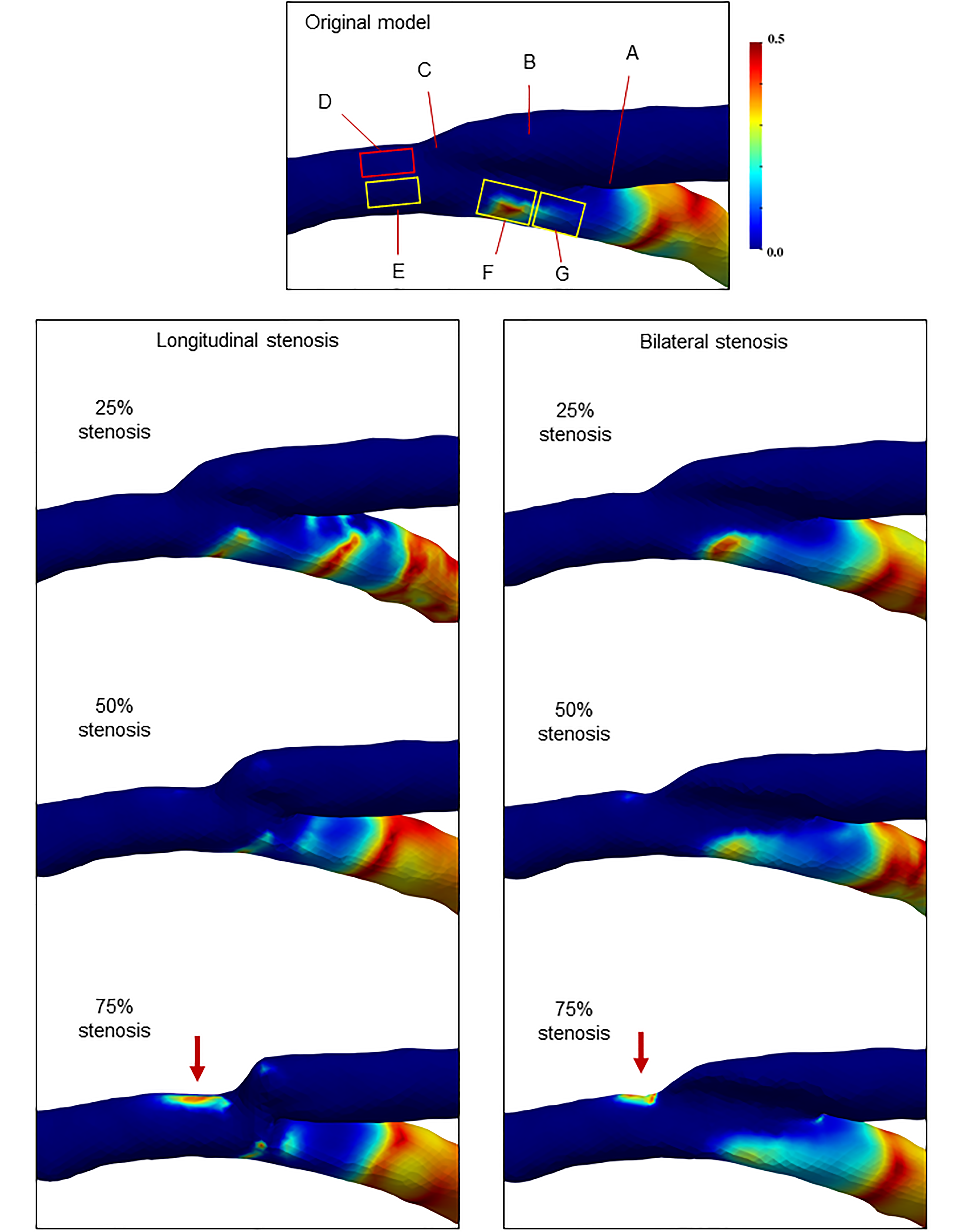

Supplement: ivaf013_Supplementary_Data [file ivaf013_supplementary_data.zip › Figure_S8__2nd_version_.tif]

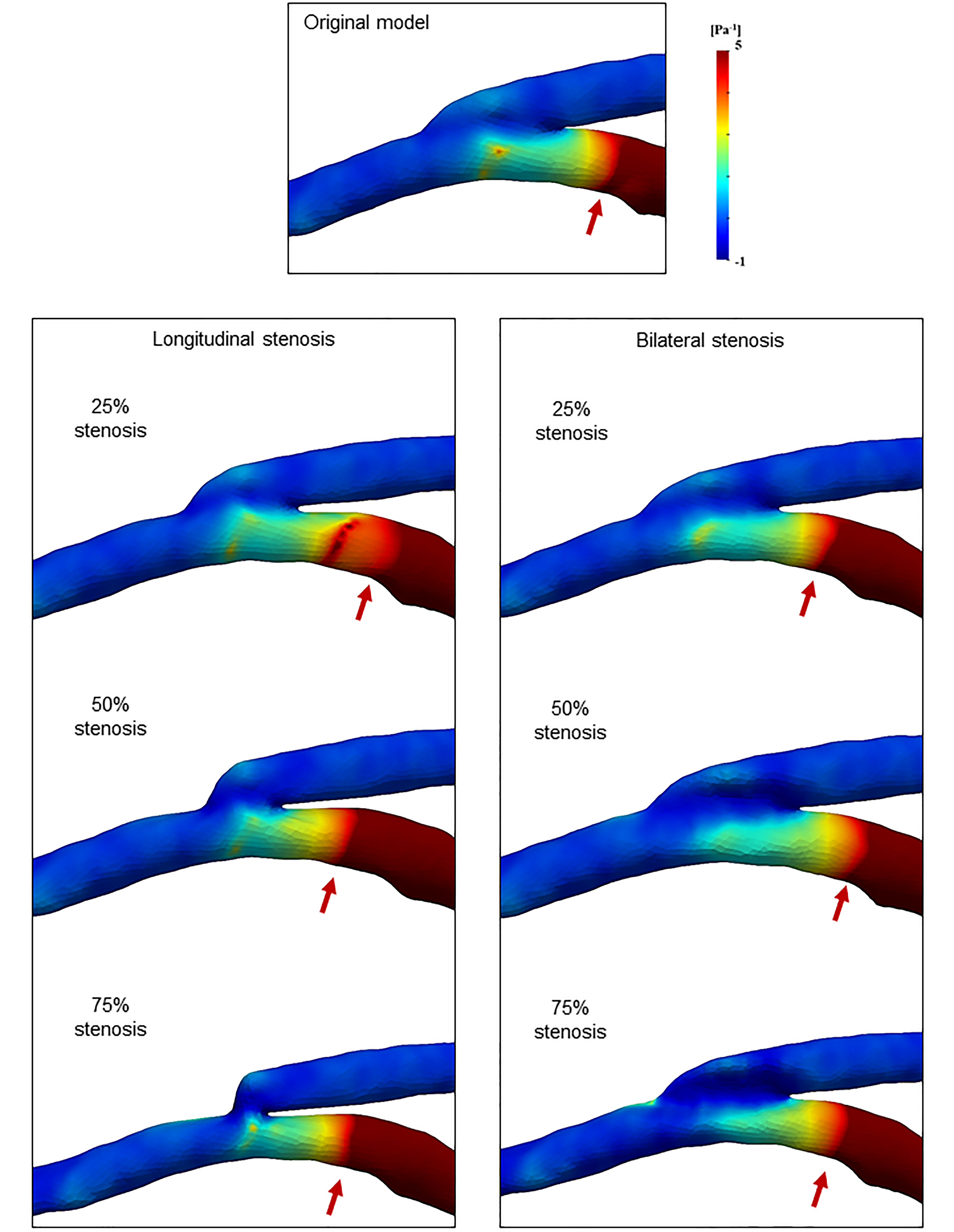

Supplement: ivaf013_Supplementary_Data [file ivaf013_supplementary_data.zip › Figure_S9__2nd_version_.TIF]

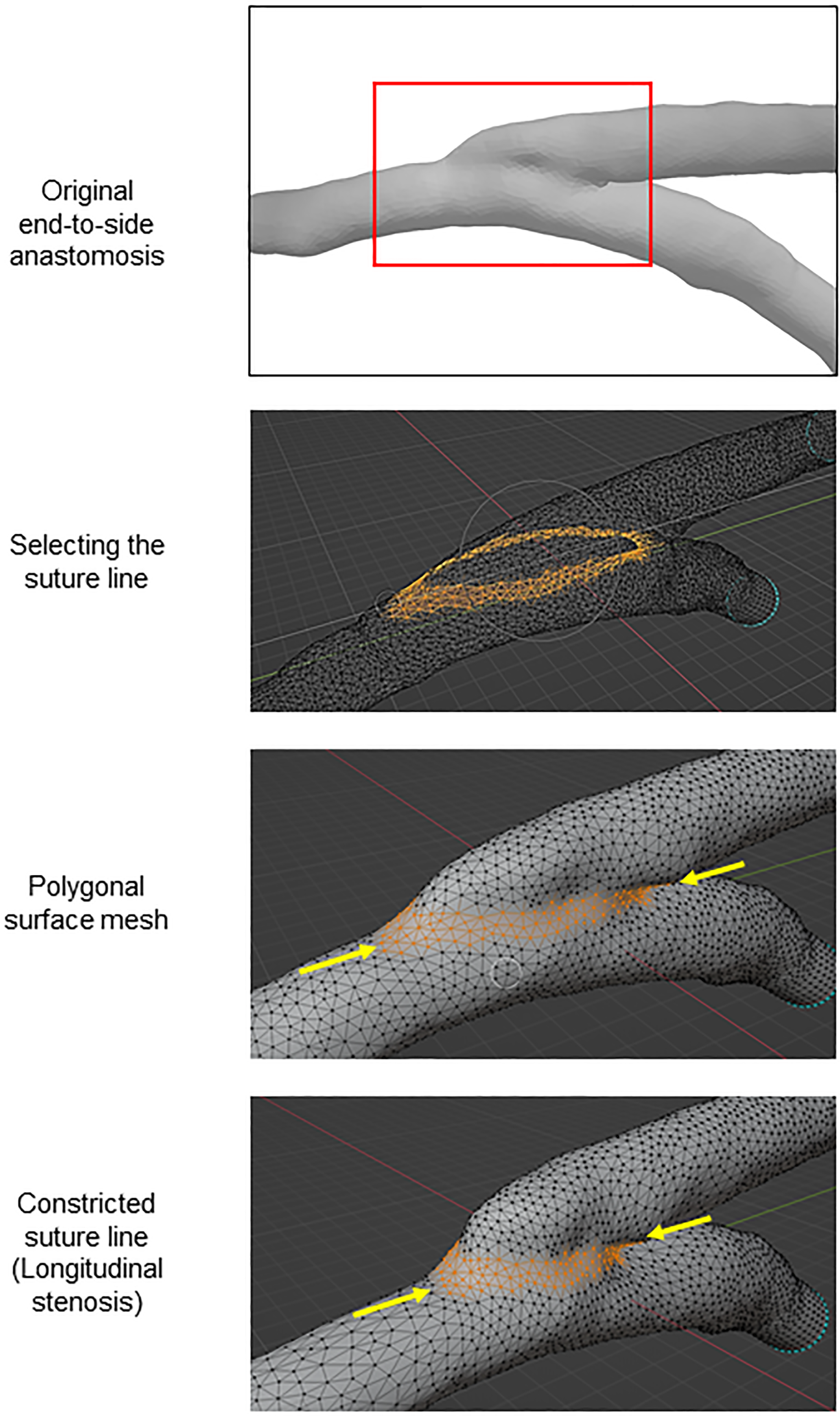

Supplement: ivaf013_Supplementary_Data [file ivaf013_supplementary_data.zip › Figure S1 (version2).TIF]

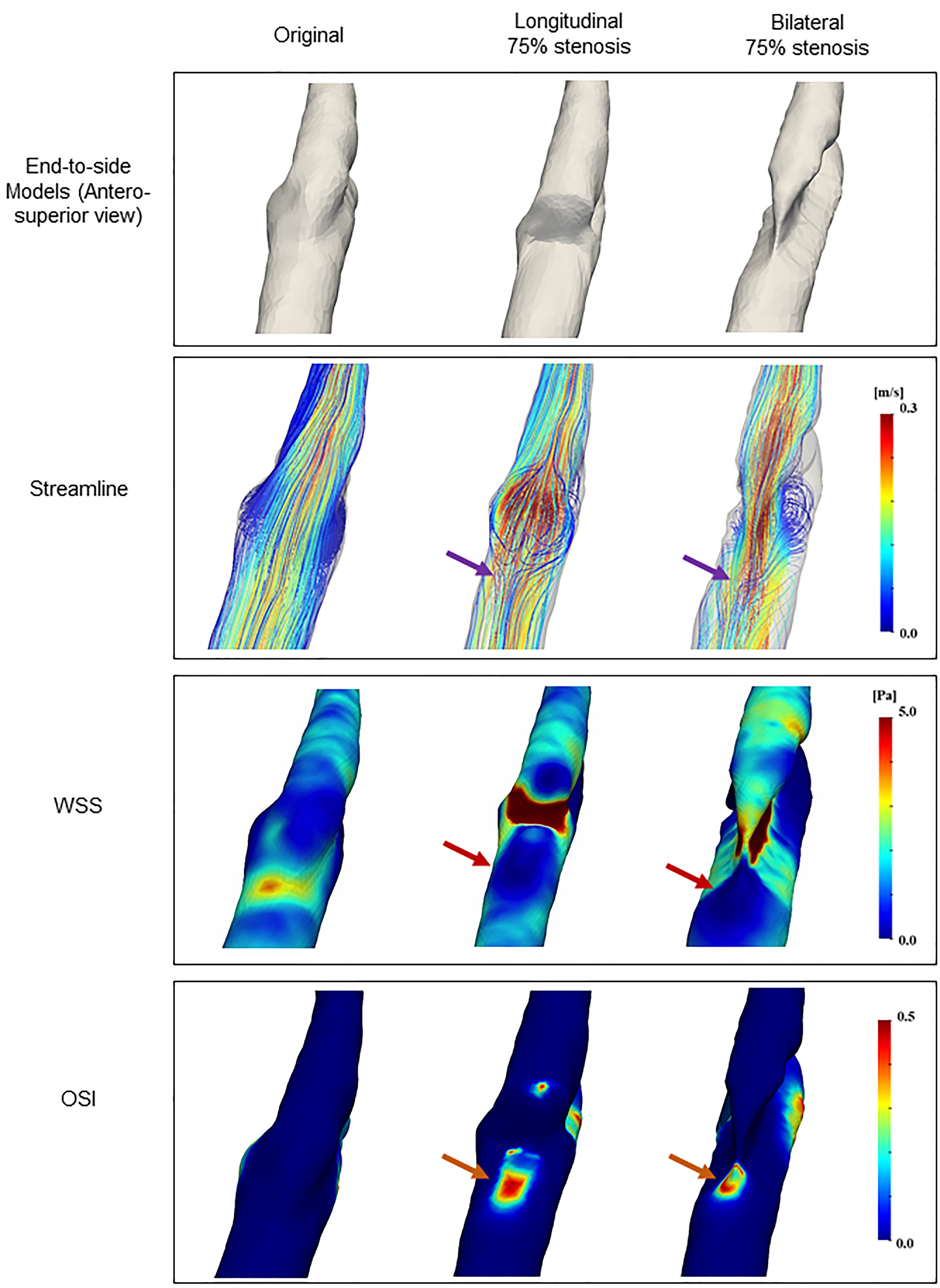

Supplement: ivaf013_Supplementary_Data [file ivaf013_supplementary_data.zip › Figure S2 (version2).TIF]

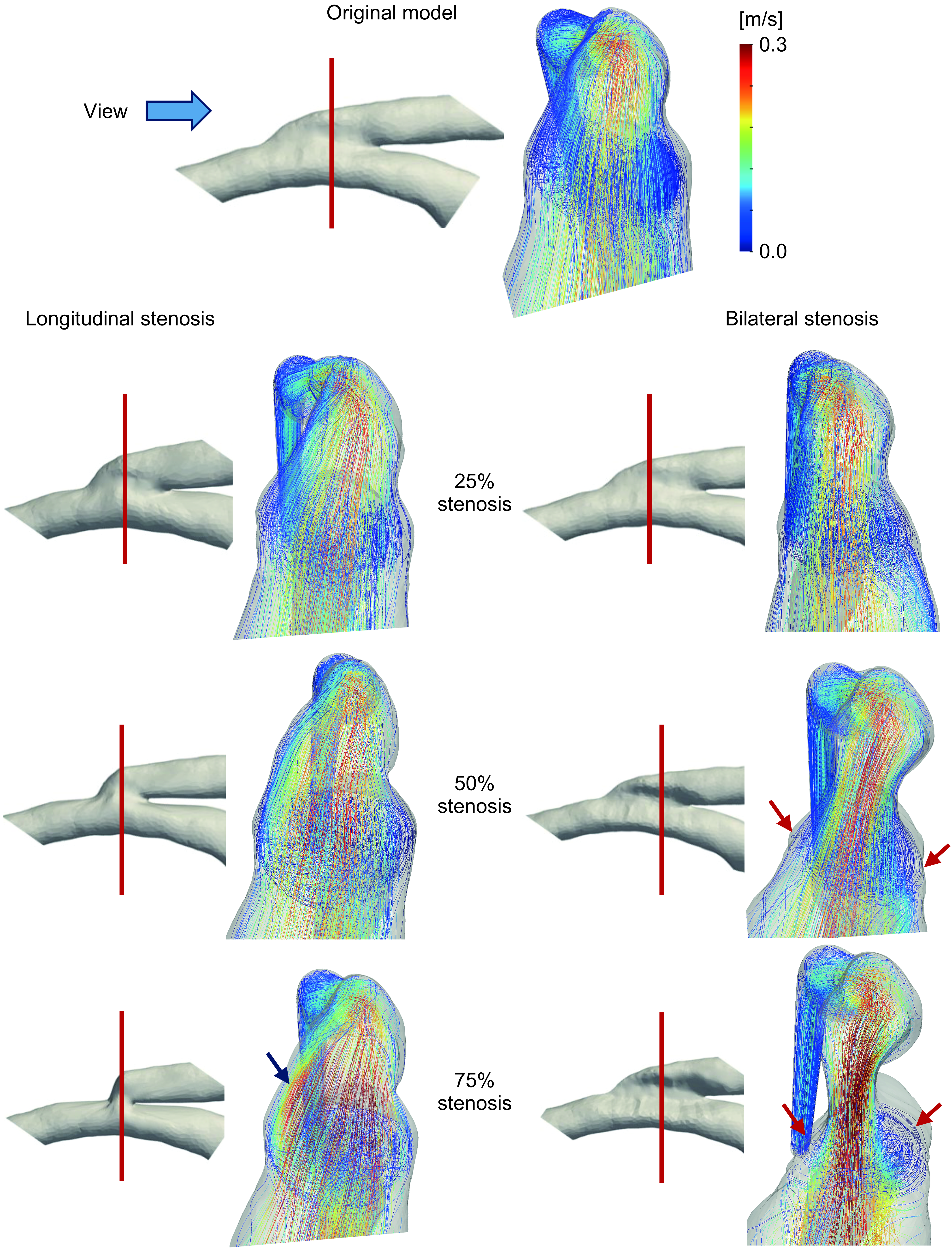

Supplement: ivaf013_Supplementary_Data [file ivaf013_supplementary_data.zip › Figure S3.tif]

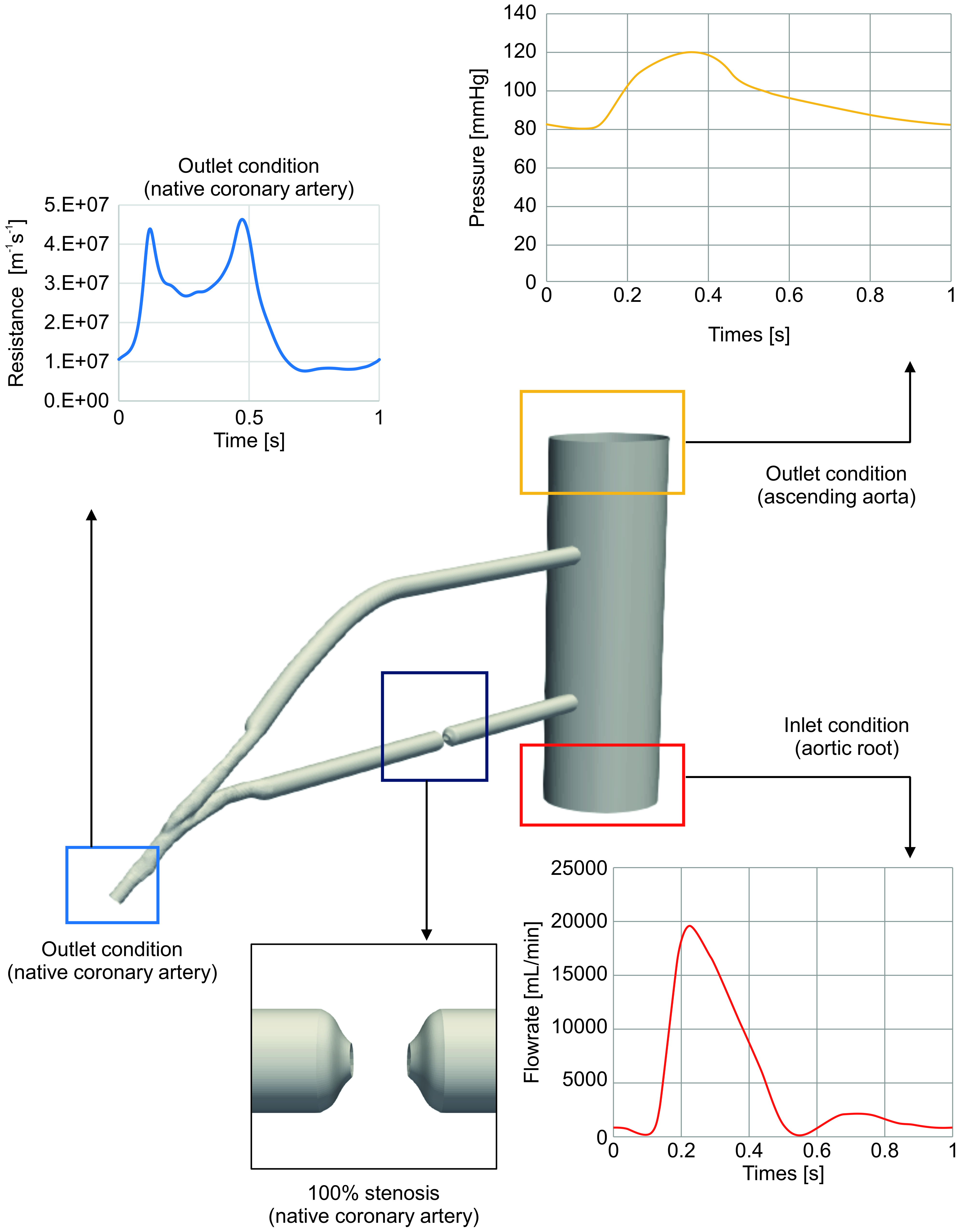

Supplement: ivaf013_Supplementary_Data [file ivaf013_supplementary_data.zip › Figure S5.tif]

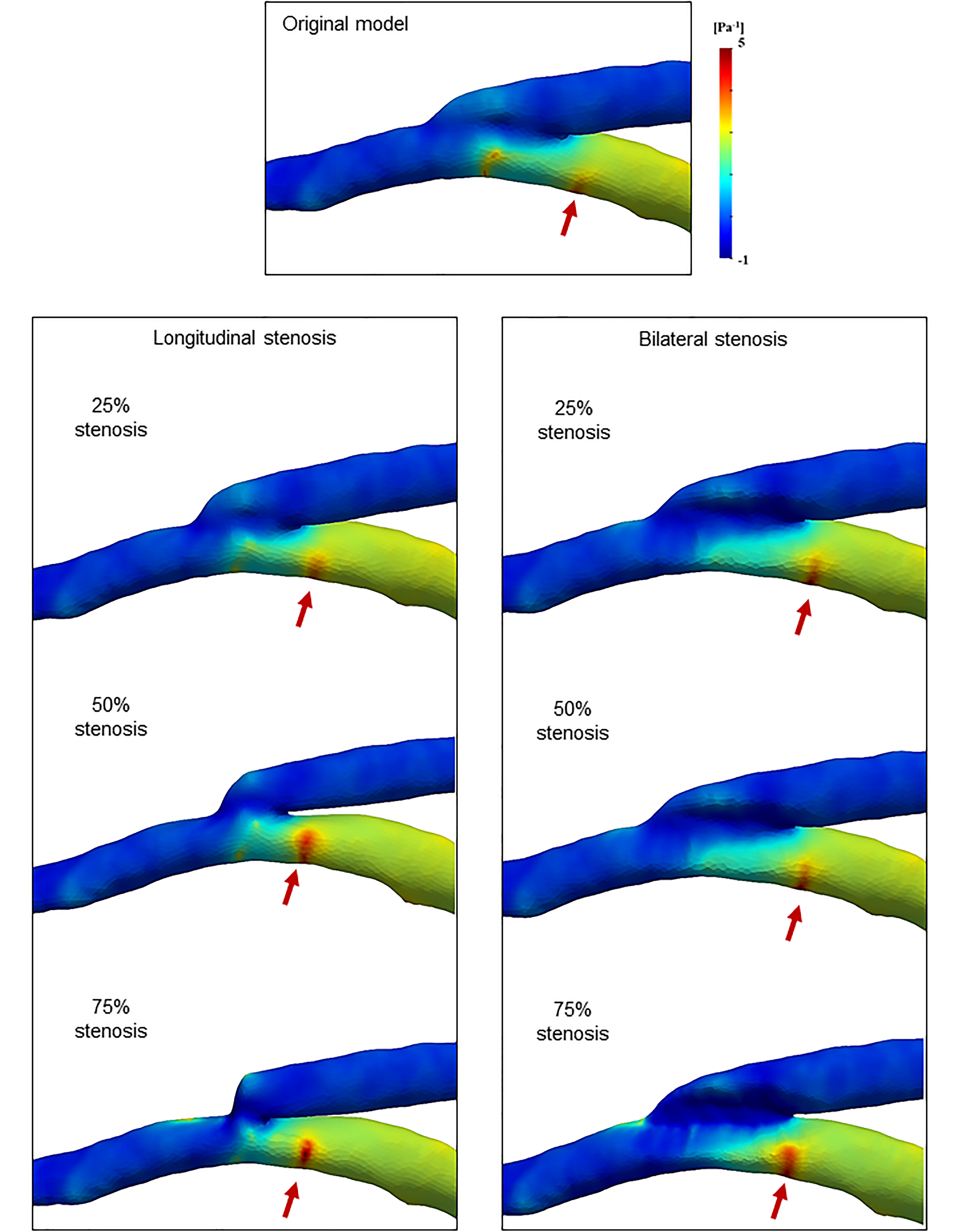

Supplement: ivaf013_Supplementary_Data [file ivaf013_supplementary_data.zip › Figure_S4__2nd_version_.tif]

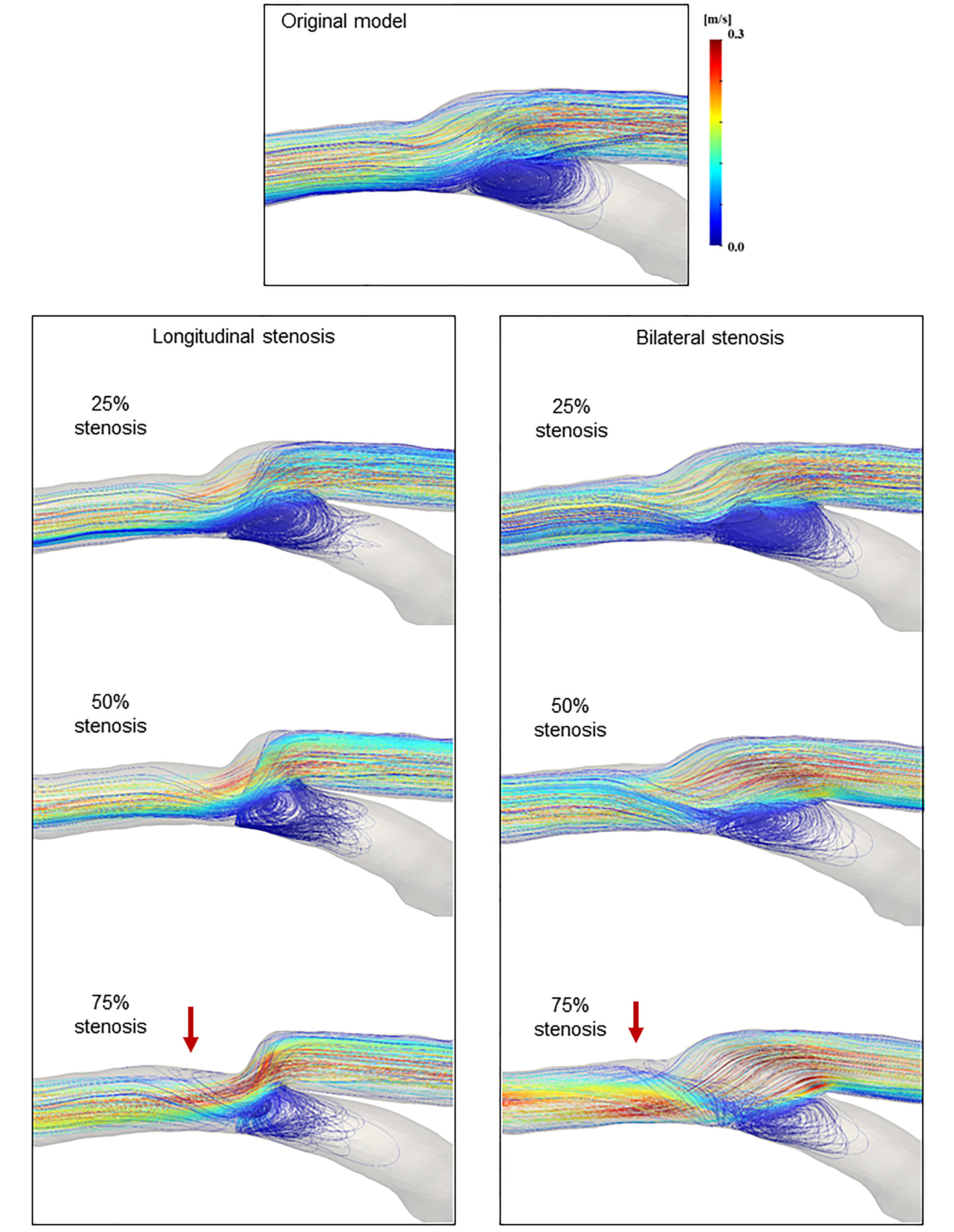

Supplement: ivaf013_Supplementary_Data [file ivaf013_supplementary_data.zip › Figure_S6__2nd_version_.TIF]
